# Revisiting Ferroelectric‐Gated Phototransistors: A Tripartite Synapse‐Inspired Approach to In‐Sensor Image Processing

**DOI:** 10.1002/adma.202503475

**Published:** 2025-07-28

**Authors:** Yubin Lee, Dong Hyun Seo, Jun Seo Lee, Jae Min Jeon, Hyung Rae Kim, Min Seok Kim, Chaehyeon Ahn, Sung‐Un An, Jihun Choi, Hyunseung Kim, Chang Kyu Jeong, Hyunseob Lim, Dong‐Ho Kang, Young Min Song

**Affiliations:** ^1^ School of Electrical Engineering and Computer Science Gwangju Institute of Science and Technology Gwangju 61005 Republic of Korea; ^2^ Department of Chemistry Gwangju Institute of Science and Technology Gwangju 61005 Republic of Korea; ^3^ Division of Advanced Materials Engineering Jeonbuk National University Jeonju Jeonbuk 54896 Republic of Korea; ^4^ Department of Energy Storage/Conversion Engineering and Hydrogen & Fuel Cell Research Center Jeonbuk National University Jeonju Jeonbuk 54896 Republic of Korea; ^5^ Department of JBNU–KIST Industry–Academia Convergence Research Jeonbuk National University Jeonju Jeonbuk 54896 Republic of Korea; ^6^ Center for Quantum Conversion Research Institute for Basic Science (IBS) Gwangju 61005 Republic of Korea; ^7^ Department of Semiconductor Engineering Gwangju Institute of Science and Technology Gwangju 61005 Republic of Korea; ^8^ AI Graduate School Gwangju Institute of Science and Technology Gwangju 61005 Republic of Korea; ^9^ School of Electrical Engineering Korea Advanced Institute of Science and Technology (KAIST) 291 Daehak‐ro, Yuseong‐gu Daejeon 34141 Republic of Korea

**Keywords:** electrical processing, ferroelectric, in‐sensor image processing, optoelectronic, phototransistor, synaptic device, tripartite synapse

## Abstract

Neuromorphic devices inspired by the tripartite synapse system offer enhanced modulation of synaptic weight via a third terminal. However, using an electrically independent terminal for memorizing and processing optical information remains unexplored. Here, a ferroelectric‐gated phototransistor (FGPT) incorporating ferroelectric polymers and organic photoactive channels is revisited for neuromorphic vision systems. It is demonstrated that partial polarization switching in the ferroelectric gate insulator enables linear control of the photoactive channel. Furthermore, the photogating effect induced by charge trapping at the ferroelectric insulator/photoactive channel interface further enhances the photonic non‐volatile (PNV) characteristics of the FGPT. This allows memorized visual information, expressed as photoconductance, to be incrementally potentiated or depressed. The modulated photoconductance fully spans the current level within the dynamic range of the device (153 dB). Finally, the feasibility of the device for all‐day face recognition is shown by in‐sensor processing of visual information obtained from unstructured environments into the pre‐trained range. This approach results in up to a ≈40% improvement in recognition accuracy.

## Graphical abstract

A ferroelectric‐gated phototransistor (FGPT) enables linear and multilevel modulation of photoconductance through partial polarization switching and photogating effects. The device achieves photonic non‐volatility over a 153 dB dynamic range and performs in‐sensor processing under unstructured illumination, demonstrating enhanced face recognition accuracy by recalibrating distorted visual inputs into a pre‐trained range.

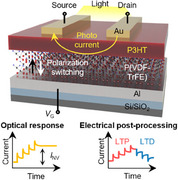

## 1 Introduction

Visual information in real‐world environments consists of multidimensional data, including light intensity, wavelength, polarization, and phase, which are inherently unstructured and lack a consistent pattern.^[^ ^1^ ^]^ The broad solar spectrum, along with varying reflection and scattering phenomena in physical environments, induces significant spatial and temporal fluctuations in light intensity. As a result, the observed light intensity range extends up to 280 dB, spanning from dim indoor to bright outdoor environments throughout the day. However, conventional complementary metal‐oxide‐semiconductor (CMOS) image sensors have a limited dynamic range of only 70 dB and suffer substantial performance degradation under low‐light conditions.^[^ ^2^, ^3^, ^4^ ^]^ Therefore, CMOS‐based vision systems encounter significant challenges in applications requiring real‐time visual information processing, such as unmanned vehicles and robotic vision systems.^[^ ^5^, ^6^ ^]^ A wide range of techniques have been proposed to overcome these limitations. Traditional methods, including high dynamic range (HDR) imaging, histogram sliding,^[^ ^7^ ^]^ stretching,^[^ ^8^ ^]^ and histogram equalization,^[^ ^9^ ^]^ primarily rely on post‐processing to enhance captured images under extreme lighting conditions. In parallel, recent advances have introduced more adaptive strategies such as event‐based sensing, transformer‐driven illumination correction, and gradient‐informed exposure control, aiming to enhance real‐time performance and robustness under complex environments.^[^ ^10^, ^11^, ^12^ ^]^ Despite these developments, most of these techniques still depend on computationally intensive operations after data acquisition and require substantial data transmission between sensing and processing units. Thus, these strategies result in limited bandwidth, high latency, and highenergy consumption due to the transmission of raw and redundant data.^[^ ^13^ ^]^ Such constraints fundamentally limit real‐time processing, particularly in unstructured environments where conditions are unpredictable.

In contrast, neuromorphic vision systems inspired by biological neural networks have emerged as a promising alternative to machine and robotic vision.^[^ ^14^, ^15^, ^16^, ^17^ ^]^ Among them, three‐terminal phototransistors have garnered attention for their ability to mimic synaptic functions. They offer effective modulation of synaptic weight, which is represented by channel conductance, to adjust or adapt to a wide range of visual information.^[^ ^18^ ^]^ In this configuration, the optical signals are inherently processed within the device, where changes in channel conductance through the gate terminal store and modulate incoming visual information.^[^ ^19^, ^20^, ^21^ ^]^ This non‐volatile photonic memory effect allows for the retention of optical data and electrical erasure, enabling key neuromorphic functions of learning, memorizing, and forgetting.^[^ ^21^, ^22^, ^23^ ^]^ Despite considerable advancements, photonic memory still faces significant challenges. Achieving multilevel conductance states with high linearity is difficult due to charge screening effects at channel‐dielectric interfaces. The photoconductance is often constrained even at high programming voltages, and the long‐term stability of optical and electrical memory properties remains challenging.^[^ ^21^ ^]^ These shortcomings lead to ambiguities in distinguishing short‐term and long‐term memory, particularly in unstructured environments where input data exceed the range of pre‐trained datasets, including light intensity and frequency variations. Addressing these issues typically requires re‐learning or reconstruction of learning datasets,^[^ ^24^, ^25^ ^]^ which increases computational costs and latency. A vision system capable of potentiation, depression, and erasure directly within the sensor would resolve these problems, enabling efficient recalibration of data and reducing computational burdens.^[^ ^26^, ^27^, ^28^ ^]^

To address these hurdles, we drew inspiration from the tripartite synapse system, which describes bidirectional communication among astrocytes, pre‐synapse, and post‐synapse.^[^ ^29^ ^]^ In the system, neuromodulators released through the astrocyte process regulate synaptic transmission and plasticity between pre‐synapse and post‐synapse to facilitate information processing, transmission, and storage. While most synaptic phototransistors can modulate photoconductance corresponding to synaptic weight through the gate terminal, their functionality is often restricted to erasure.^[^ ^21^, ^30^, ^31^ ^]^ Furthermore, to realize the visual adaptation, they require predefined channel states before sensing light signals.^[^ ^18^ ^]^ This constraint aligns more closely with a bipolar synapse system, where the gate serves as a pre‐synapse terminal rather than emulating the independent and active modulation observed in the tripartite synapse system.

Here, we introduce a ferroelectric‐gated phototransistor (FGPT) inspired by the tripartite synapse system. Unlike conventional ferroelectric field‐effect transistors (FeFETs) and phototransistors designed to achieve high non‐volatility^[^ ^32^, ^33^ ^]^ and photoresponsivity,^[^ ^34^, ^35^ ^]^ the proposed FGPT explores the potential as an in‐sensor processor by mimicking the role of neuromodulators.^[^ ^36^, ^37^ ^]^ Similar to how neuromodulators process and store synaptic information, the FGPT retains visual information for extended periods and independently adjusts memorized visual information. In this device, polyvinylidene fluoride‐trifluoroethylene (P(VDF‐TrFE)) serves as the ferroelectric gate insulator, while poly(3‐hexylthiophene) (P3HT) forms the photoactive channel that can effectively absorb light across a broad visible spectrum. The photogating effect, induced by charge trapping at the interface between the ferroelectric insulator and the photoactive channel, significantly enhances the photonic non‐volatility of the FGPT.^[^ ^38^, ^39^, ^40^ ^]^ Furthermore, partial polarization of the ferroelectric insulator modulates channel conductivity,^[^ ^41^, ^42^ ^]^ enabling gradual potentiation and depression of photoconductance. The unique partial polarization switching mechanism of the ferroelectric polymer allows for multistate modulation of photoconductance, fully spanning the dynamic range of the FGPT. Finally, we demonstrate that the FGPT can directly process unstructured lighting conditions within the sensor, maintaining compatibility with pre‐trained models without re‐learning or data reconstruction. For instance, face recognition under varying light conditions—from nighttime to sunny daylight—often suffers from distortion due to variations in light intensity. Our FGPT compensates for these nontrivial distortions by recalibrating the detected intensity within the sensory node itself, thereby ensuring reliable “all‐day” face recognition throughout the entire day.

## 2 Results and Discussion

### 2.1 In‐Sensor Image Processing Inspired by a Tripartite Synapse System

**Figure 1:**
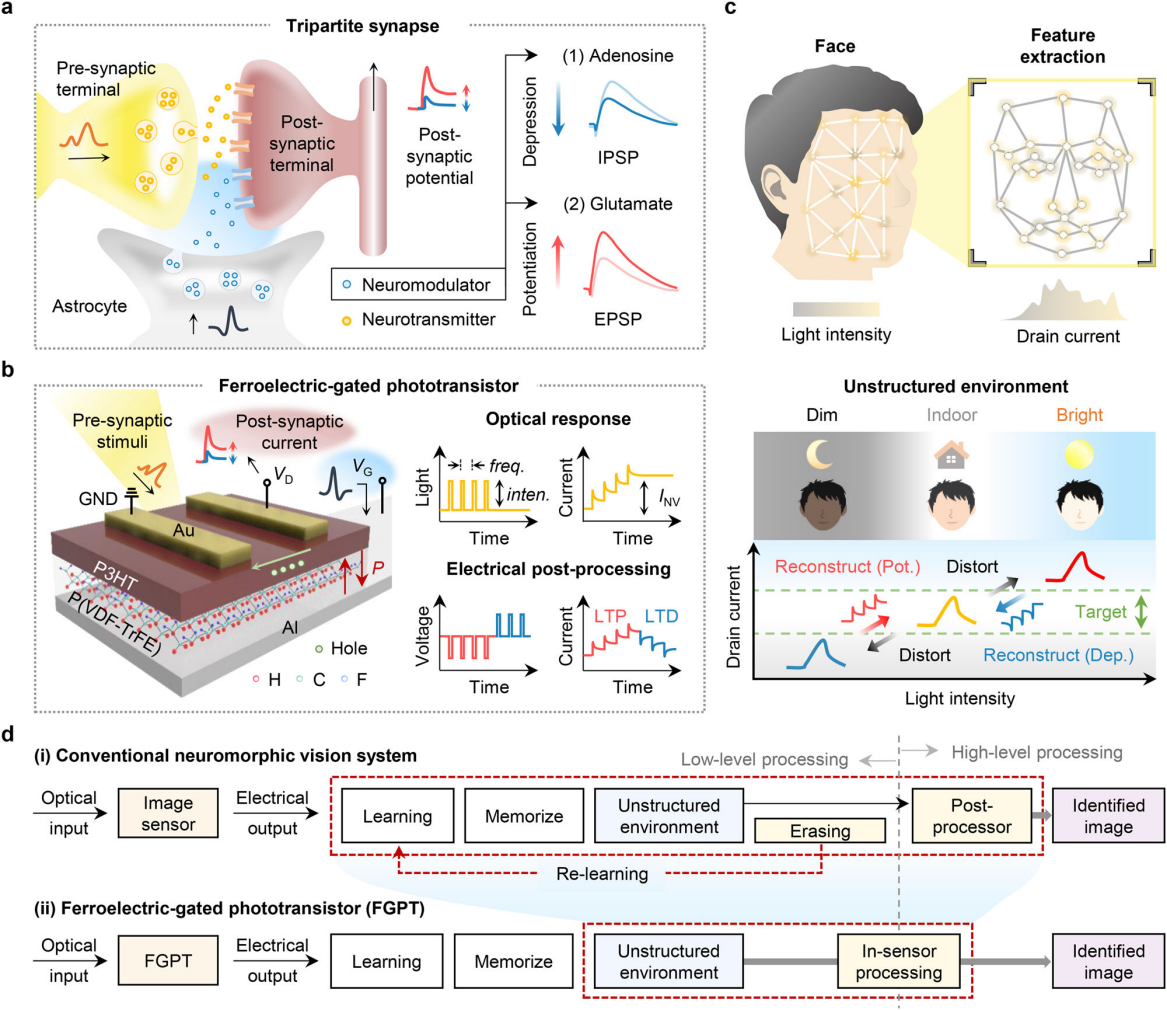
In‐sensor image processing inspired by a tripartite synapse system. a) Schematic of tripartite synapse, illustrating synaptic information processing. Adenosine induces depression, generating inhibitory post‐synaptic potentials (IPSPs), while glutamate enhances potentiation, producing excitatory post‐synaptic potentials (EPSPs). b) Schematic of the ferroelectric‐gated phototransistor (FGPT) with P3HT photoactive channel and a P(VDF‐TrFE) ferroelectric gate insulator. GND, *V*_D_, and *V*_G_ represent ground, drain voltage, and gate voltage, respectively. Pre‐synaptic stimuli (i.e., light frequency (*freq*.) or intensity (*inten*.)) are stored as nonvolatile current (*I*_NV_). Electrical post‐processing modulates polarization (*P*) to control post‐synaptic current, enabling long‐term potentiation (LTP) and long‐term depression (LTD). c) Illustration of face illumination and feature extraction (top). In an unstructured environment (i.e., dim, indoor, bright), light‐induced distortion is reconstructed to the target range using potentiation (Pot.) or depression (Dep.) pulses (bottom). d) Comparison of image recognition in i) conventional neuromorphic vision system and ii) FGPT‐based in‐sensor vision system. The FGPT directly processes unstructured environment data within the sensor, eliminating the need for re‐learning.

Recent studies have demonstrated that astrocytes in the tripartite synapse system play a direct role in processing synaptic information, which is closely linked to human long‐term memory and learning.^[^ ^43^ ^]^ As illustrated in **Figure** **1**a, neuromodulators released by astrocytes regulate synaptic transmission by inhibiting or facilitating synaptic activity, thus influencing synaptic plasticity. For example, in hippocampal synapses, astrocyte‐released adenosine and glutamate contribute to long‐term depression (LTD) and long‐term potentiation (LTP), respectively, by tuning inhibitory post‐synaptic potentials (IPSPs) and excitatory post‐synaptic potential (EPSP).^[^ ^44^ ^]^ Also, they are released when behavioral outcomes deviate from expectations, modulating dendritic spine expansion or contraction to strengthen or weaken specific memories. These biological mechanisms can be considered analogous to the adjustment of learning rates and network complexity in deep neural networks (DNNs),^[^ ^45^ ^]^ inspiring further advancements in neuromorphic devices.^[^ ^36^, ^37^ ^]^

Inspired by the tripartite synapse system, FGPT with an organic photoactive channel is proposed (Figure 1b). Unlike conventional optoelectronic synaptic devices, where synaptic weight modulation primarily focuses on erasing functions,^[^ ^46^ ^]^ the FGPT incorporates a ferroelectric gate insulator to enable independent and stable channel modulation, similar to astrocyte‐mediated neuromodulation in the tripartite synapse (Table S1, Supporting Information). Incident light serves as a pre‐synaptic stimulus, while the induced photocurrent corresponds to the post‐synaptic current. Because light intensity and frequency are stored as a non‐volatile photocurrent (*I*_NV_), the FGPT can retain optical information for extended durations. Furthermore, the stored photoconductance can be electrically modulated to achieve gradual potentiation and depression by controlling ferroelectric polarization (*P*).^[^ ^47^, ^48^ ^]^

The benefits of this approach become evident in Figure 1c, which illustrates the face‐recognition mechanism of the FGPT under unstructured lighting conditions, spanning environments from dim to bright. Conventional systems relying on light intensity often struggle with uneven or biased illumination, resulting in distorted feature extraction and requiring post‐processing or re‐learning.^[^ ^49^, ^50^ ^]^ In contrast, the FGPT directly memorizes and processes light‐intensity data as photoconductance within the device, effectively reconstructing distorted images in real time and maintaining compatibility with pre‐trained recognition models throughout the day. This in‐sensor processing confers significant advantages for robust vision systems, which typically face major challenges due to fluctuating illumination.

Finally, Figure 1d compares the FGPT with conventional neuromorphic vision systems. As illustrated in Figure 1d (i), many existing devices discard un‐trained or out‐of‐range data (akin to biological forgetting) and repeatedly re‐learn using new datasets or algorithms to adapt to unstructured environments.^[^ ^51^ ^]^ These approaches greatly increase computational cost and system complexity by requiring additional hardware and software post‐processing units.^[^ ^52^ ^]^ In contrast, Figure 1d (ii) highlights how FGPT recalibrates incoming light signals directly within the sensory node via gate‐pulse modulation, preserving the use of pre‐trained data across a wide range of lighting environments. This in‐sensor processing eliminates the need for re‐learning and substantially reduces computational overhead, laying the groundwork for more efficient and adaptive neuromorphic vision systems.

### 2.2 Optoelectronic Synaptic Characteristics of FGPT

**Figure 2:**
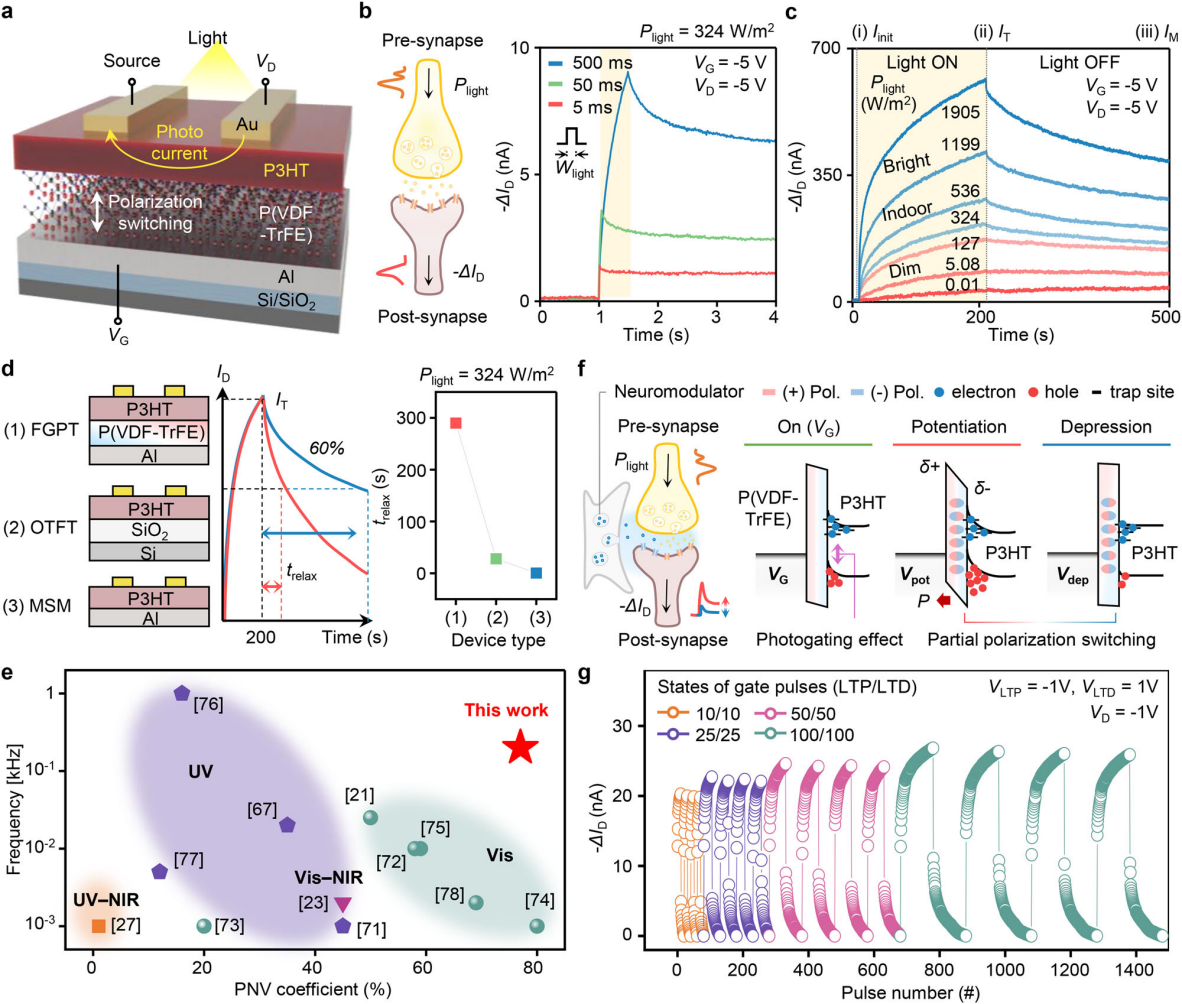
Optoelectronic and synaptic characteristics of the FGPT. a) Schematic of the FGPT structure, showing its components and functional layers. b) Photonic response characterized by drain current change (*ΔI*_D_) under different light pulse widths (*W*_light_ = 5, 50, 500 ms). c) Photonic response across seven light intensity (*P*_light_), showing transient current (*I*_T_) measured just before the light turns off and memory current (*I*_M_) recorded at 500 s. d) Relaxation times (*t*_relax_) comparison for three devices: 1) FGPT, 2) organic thin‐film transistor (OTFT), and 3) metal‐semiconductor‐metal (MSM) structure. e) Energy band diagram illustrating photogating effect and partial polarization switching in FGPT. f) Comparison of photonic non‐volatile (PNV) coefficients under different light frequencies, highlighting superior characteristics of this work. g) Long‐term potentiation (LTP) and long‐term depression (LTD) measured at various pulse sets.

As an optoelectronic synaptic device, we validate the photonic non‐volatile (PNV) effect and ferroelectric polarization switching of the FGPT. **Figure** **2**a presents the structural schematic of the FGPT, incorporating a bottom‐gate structure to maximize light absorption. Solution‐processable P(VDF‐TrFE), highly compatible with P3HT, enables stable remnant polarization at nanoscale thicknesses and supports large‐area fabrication.^[^ ^53^, ^54^, ^55^ ^]^ The P3HT organic channel, a *π*‐conjugated material, exhibits strong visible‐light absorption.^[^ ^56^, ^57^ ^]^ Aluminum (Al) gate electrodes form a stable interface with the ferroelectric polymer, supporting uniform electric field distribution and facilitating polarization switching,^[^ ^58^, ^59^ ^]^ while gold (Au) source and drain electrodes provide ohmic contact with the p‐type P3HT channel.^[^ ^60^ ^]^ Additionally, Scanning transmission electron microscope (STEM) images further confirm the detailed structural integrity of the FGPT (Figure S1, Supporting Information). Finally, the transfer characteristics of FGPT are presented in Figure S2 (Supporting Information), and the extracted FET parameters under dark conditions are summarized in Supplementary Table S2 (Supporting Information).^[^ ^47^, ^61^, ^62^ ^]^ While trap states tend to affect the FET parameters,^[^ ^63^, ^64^, ^65^ ^]^ we accepted this trade‐off to enhance the photonic nonvolatile characteristics.

A key feature of the FGPT is its electrically non‐volatile response to variations in light intensity and temporal differences. This capability allows input light information to be translated into distinct output electrical signals, mimicking the biological behaviors between pre‐ and post‐synapse.^[^ ^66^ ^]^ Figure 2b illustrates the photonic response characteristic of the FGPT under varying light pulse width (*W*_light_) at fixed light intensity (*P*_light_) of 324 W m^−2^. The ‐*ΔI*_D_ denotes the difference in drain current (*I*_D_) between the dark state and light exposure. As *W*_light_ increases from 5 to 500 ms, the transient current (*I*_T_) peaks also rise from 1.5 to 9 nA. After the light removal, the current stabilizes within 2 s in darkness, maintaining 70–75% of the *I*_T_. Even at a *W*_light_ of 5 ms, the FGPT exhibited strong PNV characteristics, confirming its high‐speed optical response. Cumulative photocurrent behavior under continuous light pulses at 50 Hz is shown in Figure S3 (Supporting Information).

Beyond temporal resolution, the FGPT also exhibits PNV with a broad dynamic range of 153 dB for dim (0.01–5.08 W m^−2^) to bright illumination (1199–1905 W m^−2^), as benchmarked against other vision systems (Figure S4, Supporting Information). The transient and memory current change under the dynamic range is shown in Figure 2c: i) The initial current (*I*_init_) is defined in dark conditions as a baseline. ii) Under 200 s of light exposure, photocurrents increase to the *I*_T_. iii) After the light removal, the current decays to a stabilized memory current (*I*_M_). The minimum detectable *I*_M_ variation of 20 nA ensures precise state differentiation with PNV effect. The gradual transition from *I*_T_ to *I*_M_ with slight attenuation resembles the biological process from short‐term memory to long‐term memory (STM to LTM). This light response remains stable for one week without notable degradation (Figure S5, Supporting Information).

Such PNV behavior is dominated by the photogating effect arising from charge trapping at the P3HT/P(VDF‐TrFE) interface. This is further supported by threshold voltage shifts and power‐law fitting analyses, which indicate that the PNV is indeed primarily governed by the photogating effect (Note S1 and Figure S6, Supporting Information).^[^ ^67^, ^68^, ^69^, ^70^ ^]^ Nevertheless, to assess the potential contribution of ferroelectric properties to PNV, we performed a structural investigation on three device configurations: 1) FGPT, 2) an organic thin‐film transistor (OTFT) with a SiO_2_ gate insulator (Au/P3HT/SiO_2_/p^++^‐Si), and 3) a metal‐semiconductor‐metal (MSM) structure with Au/P3HT/Al (Figure 2d). Devices were exposed to a uniform *P*_light_ (324 W m^−2^) for 200 s, followed by light removal. The relaxation time (*t*_relax_) is defined as the time required for photocurrent to decay to 60% of its peak value (*I*_T_) after light stimulation. While the OTFT and MSM exhibited *t*_relax_ of less than 50 s, only the FGPT demonstrated an extended *t*_relax_ of ≈300 s. These results confirm that only the ferroelectric‐gated structure exhibits distinct transient and memory current behaviors (Figure S7, Supporting Information). Such findings suggest that the ferroelectric gate insulator plays a synergistic role in enhancing PNV effect.

Enhanced PNV behavior, enabled by the synergy between the photogating effect and the ferroelectric gate insulator, show improved performance over other optical synaptic devices (Figure 2e).^[^ ^21^, ^23^, ^27^, ^67^, ^71^, ^72^, ^73^, ^74^, ^75^, ^76^, ^77^, ^78^ ^]^ The ratio of *I*_memory,3s_ to *I*_transient_ was defined as the PNV coefficient for comparative analysis, where *I*_transient_ and *I*_memory,3s_ represent the peak current immediately after the light pulse width and the current measured 3 s after the peak, respectively. The FGPT device exhibited a PNV coefficient of 77% at a 5 ms pulse width (Figure 2b), demonstrating the highest performance among the compared devices in terms of PNV coefficient relative to frequency. Detailed information is presented in Table S3 (Supporting Information). Furthermore, we quantified the photonic non‐volatility by extracting the slow decay constant (τ_2_) from the double exponential curve fitting of photocurrent decay curves, a standard retention metric.^[^ ^72^, ^79^ ^]^ The fitting results demonstrate a high level of PNV across a wide dynamic range. Notably, the slower decay characteristics and the corresponding current variation observed under higher illumination levels support the interpretation that photogenerated carriers can be trapped by interface defects.^[^ ^72^ ^]^ The τ_2_ values across various illumination intensities are provided in Figure S8 (Supporting Information).

While it contributes to PNV, the ferroelectric gate insulator plays a more central role by enabling partial polarization switching in response to gate pulses. In response to gate‐applied pulses, the partial polarization of P(VDF‐TrFE) modulates carrier accumulation and depletion, thereby altering the photocurrent (Figure 2f).^[^ ^47^ ^]^ The independent regulation of post‐synaptic responses (*I*_D_) by pre‐synaptic stimulation (light intensity) via the third terminal (ferroelectric gate insulator) is highly analogous to the tripartite synapse system. To evaluate both the LTP/LTD characteristics and their stability induced by partial polarization, various gate pulse sets with different pulse numbers were applied. Potentiation and depression pulses were set to −1 and +1 V, respectively, with a fixed pulse width of 1 ms. The device was tested over four cycles with pulse sets ranging from 10 to 100 pulses per set, resulting in a total of ≈1500 pulses. The LTP/LTD characteristic curves extracted under these conditions exhibited highly stable and repeatable behavior over the four cycles (Figure 2g). Synaptic properties, such as dynamic range and non‐linearity, analyzed under varying drain voltages (*V*_D_), are discussed in Note S2 and Figure S9 (Supporting Information).^[^ ^80^ ^]^ The energy band diagram that illustrates the combined mechanisms of the photogating effect and conductivity modulation is depicted in Figure S10 (Supporting Information).^[^ ^81^, ^82^, ^83^ ^]^

### 2.3 Electrical In‐Sensor Processing with Ferroelectric‐Gate Modulation

**Figure 3:**
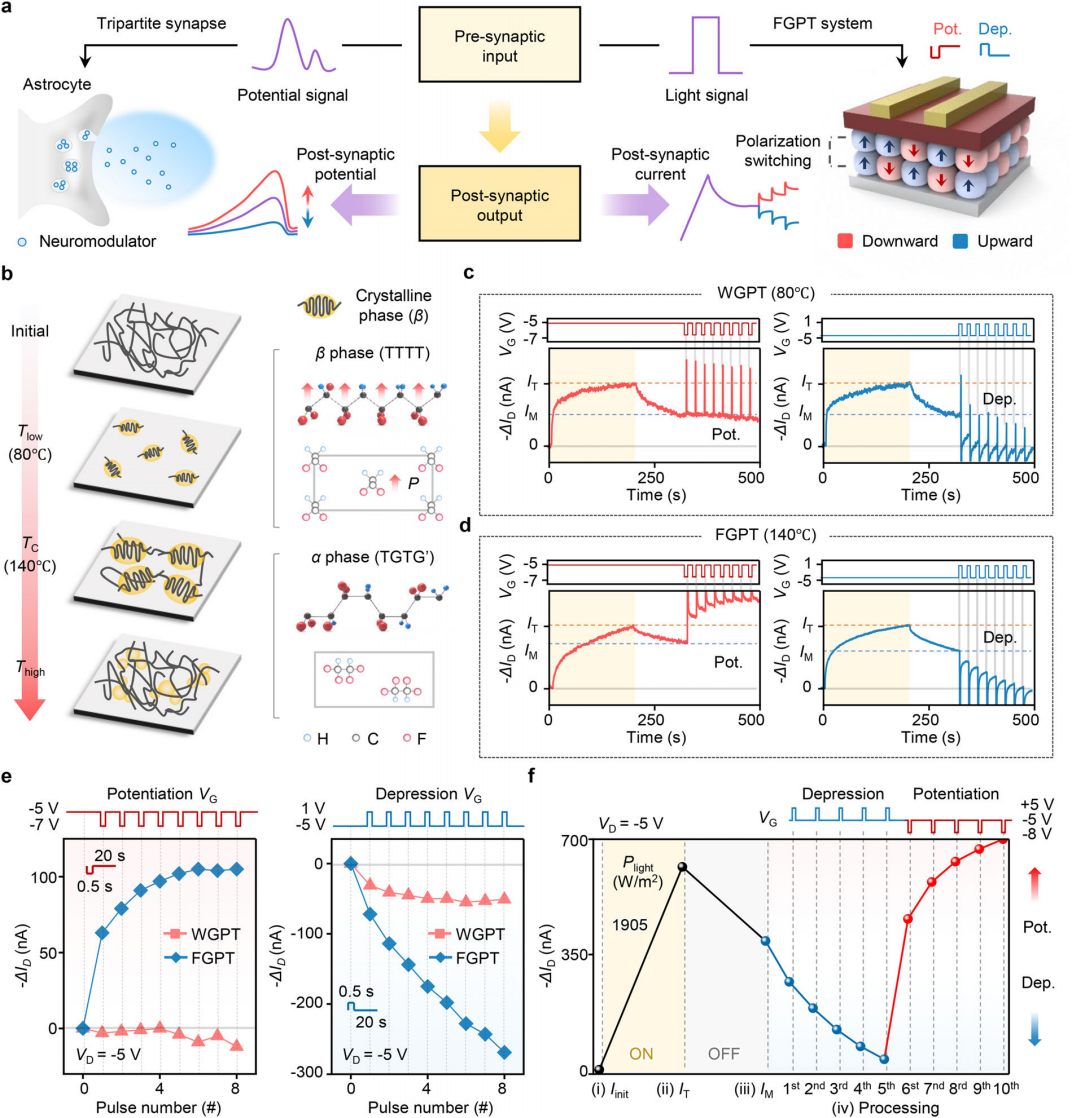
Electrical in‐sensor processing with ferroelectric‐gate modulation. a) Graphical illustrations of a tripartite synapse (left) and the FGPT system (right), where light signals dynamically control post‐synaptic current through polarization switching. b) Crystalline *β*‐phase domain size as a function of annealing temperature (left). *T*_C_, *T*_low_, and *T*_high_ denote the Curie temperature (≈140 °C), a temperature below *T*_C_, and a temperature above *T*_C_, respectively. The *β*‐phase exhibits an all‐trans (TTTT) conformation, while the *α*‐phase adopts an alternating trans‐gauche (TGTG') confirmation (right). c) Weak ferroelectric‐gated phototransistor (WGPT), annealed at 80 °C, showing rapid conductance reset in response to potentiation and depression *V*_G_ processing. d) Potentiation and depression *V*_G_ processing responses of the FGPT, demonstrating stable conductance modulation. e) Comparison of *ΔI*_D_ under potentiation and depression *V*_G_ pulses, demonstrating an extended dynamic range in FGPT (blue) compared to WGPT (pink). f) Current at each stage of sequential photonic and electrical processing: i) *I*_init_, ii) *I*_T_, iii) *I*_M_, and iv) electrical processing. The FGPT dynamically modulates memory current over a wide range (25 to 700 nA) with an optimized *V*_G_ pulse.

In biological systems, neuromodulators in tripartite synapses control synaptic transmission between pre‐ and post‐synapses.^[^ ^29^ ^]^ Inspired by this mechanism, the FGPT employs ferroelectric polarization to precisely control the memorized light information by tuning the photoconductance. As shown in **Figure** **3**a, the Al gate functions as the astrocyte, the P3HT channel represents the synaptic cleft, and the P(VDF‐TrFE) serves as the neuromodulator. Gate voltage (*V*_G_) pulses generate electric fields that induce partial polarization switching in the ferroelectric domain. This process mimics the role of neuromodulators in regulating synaptic connectivity and allows the device to modulate post‐synaptic current, analogous to the modulation of post‐synaptic potential in biological synapses.

Therefore, the ferroelectricity in P(VDF‐TrFE) plays a crucial role in controlling the photoactive channel. The ferroelectric properties were evaluated based on different annealing temperatures. P(VDF‐TrFE) thermodynamically forms a stable ferroelectric *β*‐phase near its Curie temperature (*T*_C_), where highly aligned electronegative fluorine (F) dipoles produce significant spontaneous polarization (Note S3 and Figure S11, Supporting Information).^[^ ^47^ ^]^ This polarization can be reoriented or modulated under an electric field, whereas *α*‐phases cancel the polarization vectorially due to alternating dipoles.^[^ ^84^ ^]^ Figure 3b indicates that annealing temperature significantly affects the size and distribution of *β*‐phase domains. Near *T*_C_, the *β*‐phase domains grow larger, exhibiting strong ferroelectric properties that enhance remnant polarization. Above the *T*_C_ (*T*_high_), *β*‐phase domains partially degrade, leading to the formation of nonpolar *α*‐phases.^[^ ^85^, ^86^, ^87^ ^]^

We evaluated whether the *I*_M_ can be electrically post‐processed by the ferroelectric gate insulator. Figure 3c,d illustrates the *I*_D_ behavior under *V*_G_ pulses in phototransistors with gate insulators exhibiting different ferroelectric properties. Specifically, (c) WGPT refers to devices incorporating a weak ferroelectric insulator annealed at 80 °C, resulting in smaller *β*‐phase domains and insufficient ferroelectricity. In contrast, (d) our FGPT is annealed at 140 °C, displaying larger *β*‐phase domains and stronger ferroelectricity. *V*_G_ for potentiating *I*_M_ was pulsed from −5 to −7 V, while *V*_G_ for depressing *I*_M_ was pulsed from −5 to 1 V, both for 0.5 s and repeated eight times. As shown in Figure 3c, WGPT exhibited minimal *I*_D_ response during potentiation and rapid current reset during depression, whereas FGPT maintained non‐volatile *I*_D_ with gradual and controlled modulation (Figure 3d).

Figure 3e compares electrically non‐volatile behavior based on *I*_D_ changes measured in Figure 3c,d. Only the FGPT exhibits stable non‐volatility with gradual current modulation under *V*_G_ pulses. In contrast, the WGPT shows no potentiation and a significantly lower depression current (51 nA) compared to the FGPT (270 nA). This difference arises from the strong ferroelectric polarization of FGPT, enabling partial switching at lower electric fields and efficient modulation under identical gate pulses. Negative (excitatory) pulses generate a downward electric field, enhancing polarization and increasing hole concentration at the P3HT channel, leading to the potentiation of *I*_D_. Positive (inhibitory) pulses create an upward electric field, reversing polarization and decreasing hole concentration, resulting in the depression of *I*_D_.^[^ ^88^, ^89^, ^90^ ^]^ In phototransistor structures without a ferroelectric gate insulator (OTFT), no *I*_D_ changes are observed in response to *V*_G_ pulses (Figure S12, Supporting Information).^[^ ^91^ ^]^ In addition, the application of a gate pulse induces a sharp change in *I*_D_, but the current rapidly decays without exhibiting any non‐volatile retention. These findings emphasize the critical role of ferroelectric polarization switching in FGPT for in‐sensor processing.

Finally, we demonstrate that the range of electrically post‐processed *I*_D_ fully covers the dynamic range of the FGPT and can be stepwise controlled. In Figure 3e, the FGPT was exposed to 1905 W m^−2^ light for 200 s, followed by 300 s in darkness. Under a base voltage of −5 V, five depression pulses (+5 V) and five potentiation pulses (−8 V) were applied. Sequential optical and electrical steps enable light information storage and precise modulation of *I*_D_. Optimized *V*_G_ adjusts ferroelectric polarization, dynamically tuning the current between minimum and maximum levels to achieve the desired current value (Figure S13, Supporting Information).

### 2.4 FGPT Array Demonstration for In‐Sensor Image Processing

**Figure 4:**
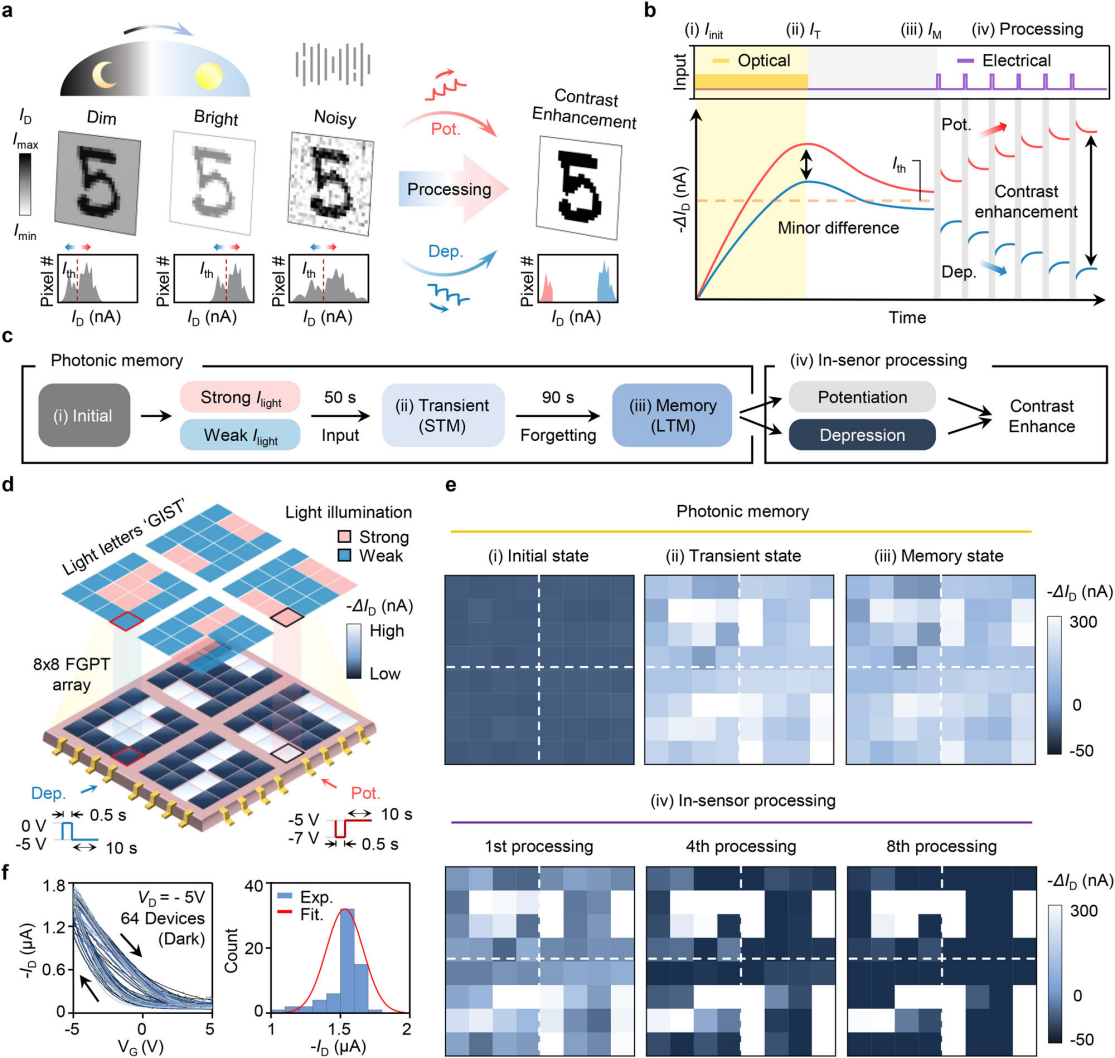
FGPT array demonstration for in‐sensor image processing. a) Contrast enhancement in unstructured environments (i.e., dim, bright, noisy) through FGPT‐based electrical processing, enabling dynamic contrast adjustment and improved image recognition. Pixel #, *I*_max_, *I*_min_, and *I*_th_ denote the number of pixels, maximum *I*_D_, minimum *I*_D_, and threshold *I*_D_, respectively. b) Schematic of in‐sensor processing, where potentiation and depression pulse based on *I*_th_​ are applied to enhance contrast by amplifying photocurrent differences. The four states are i) *I*_init_, ii) *I*_T_, iii) *I*_M_, and iv) Processing. c) Process flow from photonic memory (i.e., strong *I*_light_ and weak *I*_light_) to electrical in‐sensor processing in the FGPT system. d) Light illumination scheme for an 8 × 8 FGPT array, displaying the letters “GIST” at weak (blue) and strong (pink) intensities. Weak pixels receive depression pulses (navy), while strong pixels receive potentiation pulses (white). e) Current maps of photonic memory (top) and in‐sensor processing (bottom) demonstrate contrast enhancement. f) Transfer characteristics of 64 devices (left) and histogram of ‐*I*_D_ (Exp.), fitted with a Gaussian distribution (Fit.) measured at *V*_G_ = −5 V, *V*_D_ = −5 V (right).

Under unstructured lighting conditions, such as dim, bright, or noisy environments, the contrast between the target pattern and background significantly diminishes, reducing detection accuracy. As shown in **Figure** **4**a, in‐sensor processing based on a defined threshold current (*I*_th_) effectively separates subtle variations in light intensity while suppressing noise. This enhances contrast, allowing even minor illumination differences to be distinguished, thereby improving visual clarity. The non‐volatile characteristic of FGPT, combined with its broad dynamic range, enables this functionality. Figure 4b schematically presents the process, where the FGPT stores optical information as *I*_D_ based on light intensity and processes through electrical pulses to enhance contrast. Detailed information on the FGPT array is provided in Figure S14 (Supporting Information). When varying light intensities are detected, pixels with currents above *I*_th_ receive potentiation, while those below receive depression. This selective adjustment amplifies current differences, achieving precise modulation of current states and ensuring accurate contrast modulation, even under challenging conditions with minimal light variations.

Figure 4c outlines the overall process of the contrast enhancement in the FGPT array, which includes the photonic memory effect and in‐sensor processing. i) In the dark state, no photocurrent was detected. ii) During 50 s of light exposure, weak light was applied to the letter region, while strong light illuminated the background, generating *I*_T_. iii) After light removal, the *I*_M_ persisted for 90 s, showing non‐volatile behavior. iv) Finally, electrical pulses selectively modulated the stored data, enabling potentiation or depression. To validate the array‐level functionality, an 8  ×  8 FGPT array was fabricated as shown in Figure 4d. The contrast enhancement was demonstrated by displaying the letters ‘GIST’ using weak light (127 W m^−2^) against strong background light (536 W m^−2^). Pixels in the letter region exhibit *I*_M_ below the threshold and were modulated by depression pulses with an amplitude of +5 V. In contrast, background pixels with *I*_M_ above the threshold were modulated by potentiation pulses with an amplitude of −2 V. Both pulses were applied at a base voltage of −5 V, with a duration of 0.5 s, and were repeated 8 times at 10 s intervals.

Figure 4e illustrates the photonic memory effect and in‐sensor processing in response to optical and electrical inputs, visualized as a current map displaying the “GIST” pattern. Each pixel represents an FGPT device, with colors indicating *I*_D_ variations. i) In the dark state, no discernible pattern is observed. ii) Under illumination, a faint “GIST” pattern appears as the *I*_T_ map, corresponding to weak and strong optical inputs. iii) After light removal, the *I*_T_ slightly diminishes and stabilizes as *I*_M_, retaining optical information with minimal decay. The strong optical input produces an *I*_T_ of 310 nA, which stabilizes at 245 nA, while the weak input generates 154 nA, stabilizing at 128 nA. Although both *I*_T_ and *I*_M_ exhibit differences corresponding to light intensity, the relatively small current difference indicates a limitation in accurately distinguishing between strong and weak inputs. iv) To address this, in‐sensor processing applied successive electrical pulses. By setting the *I*_M_ threshold to 200 nA, devices above the threshold received potentiation pulses, whereas those below‐received depression pulses. After 8 pulses, the current difference between strong and weak inputs increased significantly, from 117 to 405 nA, demonstrating enhanced contrast. This specific *I*_M_ threshold setting highlights the potential to automate contrast enhancement. The statistical reproducibility of this behavior across multiple devices confirms consistent modulation (Figure S15, Supporting Information). All measured photocurrent data from the 8  ×  8 FGPT array experiment is provided in Figure S16 (Supporting Information).

Finally, we confirm the uniformity of the FGPT array. Figure 4f presents the transfer curves of all devices within the array and the corresponding *I*_D_ histogram measured at a *V*_G_ of −5 V. The clockwise hysteresis loop verifies stable ferroelectric properties, and a Gaussian distribution of *I*_D_ further supports the uniformity of the array.

### 2.5 All‐Day Face Recognition under Unstructured Environments

**Figure 5:**
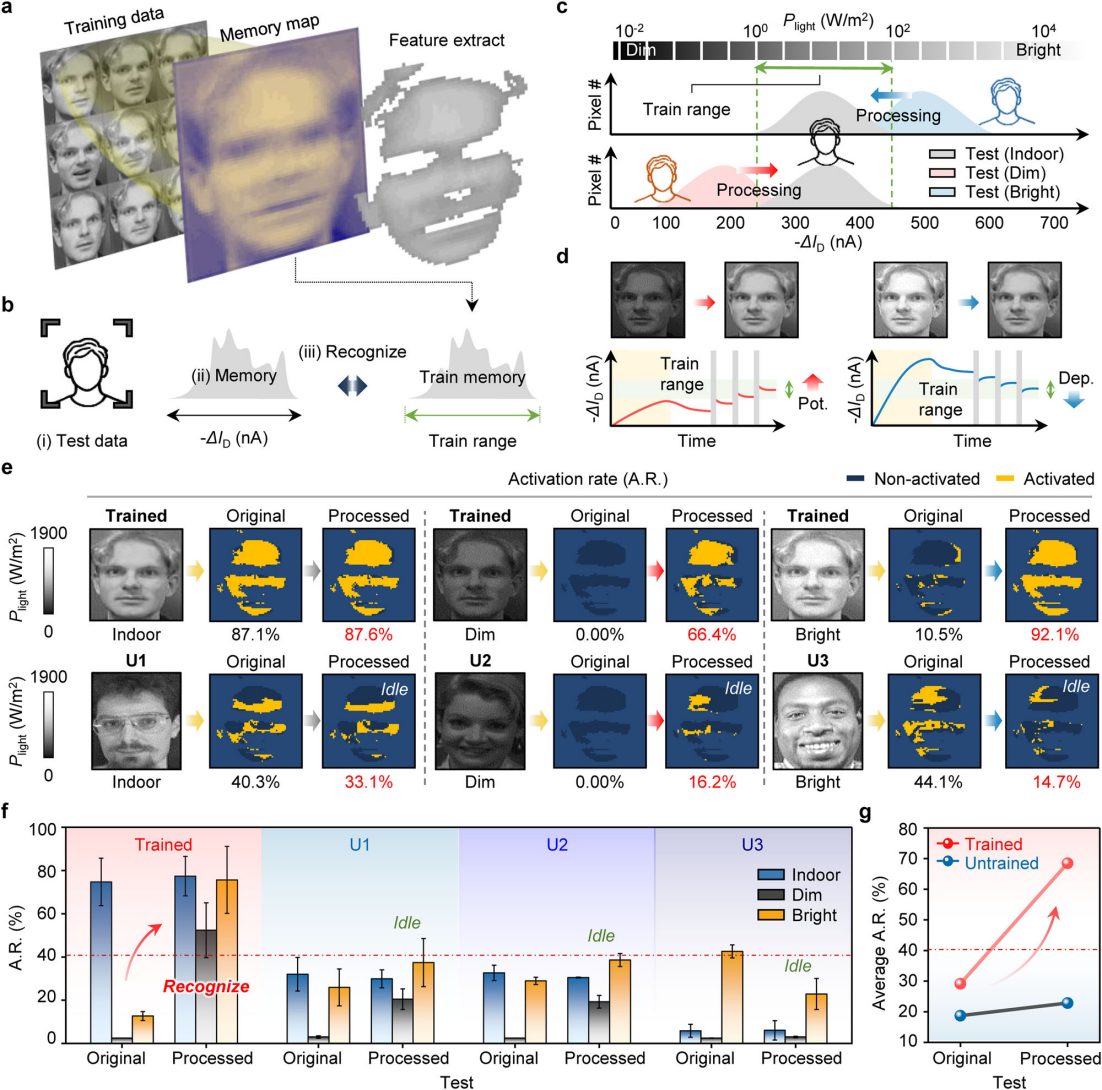
All‐day face recognition under unstructured environments. a) Training framework using a 64  ×  64 FGPT array, where memory current maps and feature masks are generated. b) The recognition process consists of i) test image projection, ii) memory current generation, and iii) pixel activation analysis. c) In‐sensor processing aligns test memory currents with the training range under different *P*_light_ (i.e., dim, indoor, bright). d) Current adjustment is performed using potentiation (left) and depression (right) pulses to match the training range. e) Activation rates (A.R.) before and after processing for trained and untrained (i.e., U1, U2, U3) faces under different lighting conditions. Non‐activated pixels are shown in navy, while activated pixels are shown in yellow. f) Comparison of A.R. before (i.e., Original) and after processing (Processed) for trained and untrained (i.e., U1, U2, U3) cases. Indoor is represented in blue, dim in navy, and bright in orange. g) Average A.R. shows a significant increase in trained data (pink) compared to untrained data (blue) demonstrating the reliability of the framework.

The in‐sensor capabilities of the FGPT were evaluated within a face recognition framework for all‐day operations.^[^ ^21^ ^]^ As shown in **Figure** **5**a, ten grayscale face images of a single individual were prepared, each featuring distinct facial expressions and orientations.^[^ ^92^ ^]^ Nine images served as the training dataset, while one was retained for testing. The training data is projected onto a 64  ×  64 FGPT array, where the optical information from each pixel is converted into memory current based on light intensity (Figure S17, Supporting Information). Upon projection, the FGPT array generates an average memory current map and establishes a training range based on the minimum and maximum current values. From this current map, feature masks were extracted at the top 75%, 50%, and 25% of the training range to accentuate facial contours and enhance recognition accuracy. Figure 5b outlines the test procedure in three steps: i) projecting the test image onto the device, ii) generating a test memory current map, and iii) comparing the test currents at each pixel to the feature‐mask currents. A pixel activates if its current lies within a specific tolerance range (7%) of the corresponding training memory current; otherwise, it remains inactive. The tolerance value was systematically optimized to minimize noise‐related errors across varied facial orientations and lighting conditions. The test data is classified as a match if 40% or more of its pixels exceed the activation threshold.

To address diverse lighting conditions, our system can modulate each pixel's current via in‐sensor processing, thereby mitigating illumination distortions and improving recognition accuracy (Figure 5c). The currents that initially exceed or fall below the training range are readjusted using gate‐voltage pulses that induce potentiation or depression of the channel conductance. Before selecting these *V*_G_, we verified that identical pulse conditions result in uniform current changes regardless of illumination levels (Figure S18, Supporting Information). We then fine‐tuned the pulse parameters experimentally and employed a curve fitting to establish an electrical processing map (Figure S19 and Table S4, Supporting Information). By applying optimized pulses derived from the processing map, the memory current is precisely adjusted to the desired level. As depicted in Figure 5d, a depression pulse is applied when the current is above the training range, whereas a potentiation pulse is applied when it is below. This approach ensures reliable and accurate recognition even in environments significantly different from the training lighting conditions (Note S4 and Figures S20–S22, Supporting Information).

Figure 5e demonstrates the activation rate (A.R.) for both trained and untrained faces under indoor, dim, and bright conditions. For trained faces in indoor conditions, 87.1% of pixels were activated. Under dim conditions, the initial A.R. dropped significantly to 0.00%, due to insufficient current response. However, in‐sensor processing adjusts the test memory current to match the training range, improving the A.R. to 66.4%. Similarly, under bright conditions, the initial A.R. was 10.5% due to excessive current. After processing, the rate significantly improved to 92.1%, highlighting effective adaptation to different lighting conditions (Figure S23, Supporting Information). In contrast, untrained faces (U1, U2, U3) exhibit relatively low A.R., ranging from 40.3% to 33.1% under indoor conditions, from 0.00% to 16.2% in dim conditions, and from 44.1% to 14.7% in bright conditions after processing (Figure S24, Supporting Information). This confirms that even after processing, untrained faces are consistently classified as non‐matching individuals. Notably, the additional reduction in activation for bright‐light untrained faces further confirms non‐matching results.

Additional data measurements and analyses highlight the importance of FGPT in various environments. Figure 5f compares the A.R. for trained and untrained data before and after processing under varying light conditions. Untrained data remained idle, while trained data initially failed to recognize but showed significant improvement after processing. Figure 5g summarizes the average recognition accuracy improvement, with trained individuals increasing from 29% to 68%, while untrained individuals remain below the 40% recognition threshold. Overall, these results validate the adaptability of our FGPT system in ensuring consistent face recognition across diverse lighting conditions, highlighting its effectiveness for reliable all‐day operation (Figure S25, Supporting Information). By enabling direct sensing and adjustment of light intensity at the hardware level, the FGPT can be combined with advanced computational algorithms to maximize recognition accuracy and enable complex image processing. In the Supporting Information, we demonstrate that integrating FGPT‐based preprocessing with a convolutional neural network (CNN) achieves more sophisticated image recognition under unstructured environments (Note S5 and Figure S26, Supporting Information).

## 3 Conclusion

Unstructured environments often lead to light information distortion, disrupting real‐time decision‐making and imaging in machine and robotic vision systems. The FGPT inspired by the tripartite synapse system, offers a promising solution by leveraging electrical and optical non‐volatility to retain light information as photoconductance over extended durations. Polarization control within the gate insulator facilitates independent and active in‐sensor processing, enabling the recalibration of unstructured data into the learned range. This approach removes the reliance on complex post‐processing and reduces computational overhead, advancing visual information processing capabilities.

Our FGPT employs phototransistor structures incorporating ferroelectric polymers and organic photoactive channels, which demonstrate outstanding electrical and optical non‐volatility. Linear modulation of channel conductivity was achieved through the partial polarization switching mechanism of the ferroelectric material. Neuromorphic behavior, characterized by LTP/LTD, displayed a non‐linearity value of ≈−3.26/−0.89 (*NL*_LTP_/*NL*_LTD_). Furthermore, the charge trapping effect and high dielectric constant of the ferroelectric insulator significantly enhanced the PNV characteristic of the photoactive channel, resulting in an increased *t*_relax_ of approximately ten times compared to conventional OTFT structures. Gradual potentiation and depression of photoconductance, enabled by linear control and non‐volatility, spanned the full dynamic range of ≈153 dB. The recalibration of light intensity data across a wide spectrum, from nighttime to bright daylight, into the pre‐trained range facilitated reliable all‐day face recognition and improved recognition accuracy compared to existing systems.

The investigation into ferroelectric phototransistors underscores their potential to integrate photonic and electronic non‐volatile memory, achieving advanced visual information processing through in‐sensor modulation of photoconductance. Future advancements may leverage the benefits of solution‐processable polymers to enable the fabrication of sensor arrays on large‐area and flexible substrates,^[^ ^93^, ^94^ ^]^ along with integration into curved optical systems for wide field‐of‐view (FoV) imaging.^[^ ^95^, ^96^ ^]^ Moreover, ferroelectric gate optimization enables spectral extension beyond the visible range, improved tolerance to optical disturbances, and expanded functionality in phase and polarization domains, while also allowing the implementation of spiking neural network (SNN) through integration with neuron devices.^[^ ^97^ ^]^

## 4 Experimental Section

### Materials and Chemicals

P(VDF‐TrFE) 70:30 mol% copolymer powder (Piezotech) was dissolved in N, N‐Dimethylformamide (DMF, Sigma–Aldrich) at a concentration of 4 wt.%. Regioregular Poly(3‐hexylthiophene‐2,5‐diyl) (P3HT, Rieke Metals) was dissolved in chloroform (Sigma–Aldrich) at a concentration of 0.5 wt.% and ultra‐sonicated at 50 °C before use. Silicon substrates with a 300 nm SiO_2_ layer, (Waferbiz) and Pt wafers (Pt 150 nm/Ti 10 nm/SiO_2_ 300 nm on a p‐type Si, GMEK) were used as platforms for device fabrication. All chemicals were used as received without further purification unless otherwise specified.

### Fabrication of Ferroelectric‐Gated Phototransistor (FGPT)

The device was fabricated on a SiO_2_ (300 nm)/Si substrate, which was first coated with a 5 nm Ti adhesion layer, followed by a 45 nm Al gate electrode deposited via electron beam evaporation (KVE‐E2000, Korea Vacuum Tech). A P(VDF‐TrFE) layer was then spin‐coated onto the Al surface at 3000 rpm for 30 s under ambient conditions, resulting in a thickness of ≈70 nm. The P(VDF‐TrFE) film was subsequently annealed at 140 °C for 2 h to remove residual DMF and improve the ferroelectric properties of the film by inducing *β*‐phase formation. Following this, a 50 nm p‐type P3HT semiconductor channel layer was spin‐coated onto the P(VDF‐TrFE) layer at 3000 rpm for 30 s under ambient conditions and annealed at 120 °C for 1 h to ensure complete solvent removal. Finally, a ≈70 nm‐thick Au layer was deposited using a thermal evaporator (SCEN‐TH‐3CH, Scientific Engineering) with a shadow mask. The formed channel length and width are 100 and 1000 µm, respectively.^[^ ^53^ ^]^

### Device Characteristic

To confirm the presence of the ferroelectric *β*‐phase, which is essential for ferroelectric properties, the copolymer solution was spin‐coated onto a SiO_2_ (300 nm)/Si substrate. The substrate was then annealed at various temperatures: 25, 80, 130, 140, 150, 160, and 170 °C. XRD measurements were conducted to detect the *β*‐phase peak near 20° using a powder X‐ray diffractometer (SmartLab, Rigaku) with a scan range of 10° to 30°, a step size of 0.01°, and a scan speed of 5°/min. For poling current measurements, an MFM structure was fabricated by spin‐coating a P(VDF‐TrFE) layer onto Pt wafers as the bottom electrode, followed by a 50 nm Au top electrode deposition using a thermal evaporator. For P–E curve measurements, MFM capacitors with Au bottom electrodes were fabricated by spin‐coating P(VDF‐TrFE) at 500 rpm for 30 s, followed by annealing and deposition of a 50 nm Au top electrode. Measurements were conducted using a polarization test system (PolyK, PolyK Technologies) with a 0.1 s signal period. The capacitor area and dielectric thickness were 7.8 × 10⁻⁷ m^2^ and 210 nm, respectively, and 1.0 µm V^−1^ scaling for electric field calculation. Notably, well‐defined P–E hysteresis loops are obtained without pre‐polling process. To verify the light absorption characteristics of the P3HT layer, the absorption spectra of the fabricated samples were measured with a UV–vis–NIR spectrometer (V‐770 EX, Jasco). The thickness of each layer was confirmed by Transmission Electron Microscopy (300 kV, Tecnai G2 F30 S‐Twin, FEI).

### Electrical and Optical Measurement Techniques

All electrical measurements, including poling current, transfer curves, and *I*–*V* characteristics, were conducted using a Precision Source/Measure Unit (B2912B, Keysight). For optical measurements, the devices were exposed to warm white LED illumination (3000 K, MWWHLP2, Thorlabs) with incident power regulated by an LED driver (LEDD1B, Thorlabs). Light frequency and pulse width were controlled using a waveform generator (33500B, Keysight) to ensure precise modulation of light conditions.

### Array‐Based Demonstration and Real‐Time Photocurrent Measurement

The array‐based setup was configured for real‐time measurement of photocurrents from the 8  ×  8 FGPT array. For this demonstration, the letters “GIST” were projected onto the array under weak illumination intensity (127 W m^−2^) against a high‐intensity background light (536 W m^−2^). A drain voltage and gate voltage of −5 V were applied during measurements. Gate pulses (base voltage of −5 V, with a 0.5 s duration and 10 s intervals) modulated the memory states. The potentiation pulse with an amplitude of −2 V and the depression pulse with an amplitude of +5 V effectively controlled the memory characteristics.

### Face Recognition Simulation

A Python‐based approach with open‐source libraries for image processing, numerical modeling, and visualization was developed to simulate face recognition under diverse lighting conditions. The simulation modeled memory and transient currents in response to various light intensities, reflecting the behavior of the device under different illumination levels. Grayscale images were adjusted to a 64  ×  64 sensor array, normalized, and scaled to represent absolute light intensities up to 2000 W m^−2^. The light intensity at each pixel was mapped to memory current values using polynomial fits based on experimental memory and transient current data. This approach captured the light‐current relationship across various illumination ranges. Each image was projected onto a simulated sensor array, generating a memory map representing current distributions influenced by light intensity. Recognition accuracy was evaluated through histograms and 3D plots, with test image intensities compared against the training memory range for alignment and recognition assessment.

## Conflict of Interest

The authors declare no conflict of interest.

## Author Contributions

Y.L., D.H.S., J.S.L., and J.M.J. contributed equally to this work. D.H.S., Y.L., J.M.J., J.S.L., Y.M.S., and D.‐H.K. conceived the work. D.H.S., Y.L., J.S.L., and J.M.J. designed the experiments and analyzed the data. J.S.L., Y.L., J.M.J., D.H.S., and C.A. fabricated devices. Y.L., J.S.L., D.H.S., J.M.J., J.C., H.K., and S.‐U.A. performed the measurements. Y.L., D.H.S., J.M.J., and J.S.L. prepared the manuscript. Y.M.S., D.‐H.K., H.R.K., M.S.K., C.K.J., and H.L. revised the manuscript. Y.M.S. and D.‐H.K. guided the entire project.

## Supporting information



Supporting Information

## Data Availability

The data that support the findings of this study are available from the corresponding author upon reasonable request.
