# Supplementary material for: Revisiting Ferroelectric‐Gated Phototransistors: A Tripartite Synapse‐Inspired Approach to In‐Sensor Image Processing
Source: Adv Mater. 2025 Jul 28;38(1):e03475. doi: 10.1002/adma.202503475 (PMC12759217; doi:10.1002/adma.202503475)
Supplement: Supplementary file 1 — Supporting Information [file ADMA-38-e03475-s001.docx]

Supporting Information

**Revisiting Ferroelectric-Gated Phototransistors: A Tripartite Synapse-Inspired Approach to In-Sensor Image Processing**

*Yubin Lee, Dong Hyun Seo, Jun Seo Lee, Jae Min Jeon, Hyung Rae Kim, Min Seok Kim, Chaehyeon Ahn, Sung-Un An, Jihun Choi, Hyunseung Kim, Chang Kyu Jeong, Hyunseob Lim, Dong-Ho Kang,^*^ and Young Min Song^*^*

**Supplementary Note 1. Photonic non-volatility induced by the photogating effect**

One of the key characteristics of the ferroelectric-gated phototransistor (FGPT) device is the photonic non-volatile effect, which can be attributed to trap sites. Such trap states lead to the dominance of the photogating effect in the device’s conduction mechanism. To verify the presence of trap sites in the FGPT device, *I*_D_-*V*_G_ transfer curves were measured under both illuminated and dark conditions. A positive shift in the threshold voltage (*V*_T_) from –1.6 V to 1.3 V was observed in the transfer curve (**Figure S6a, Supporting Information**). Considering that P3HT is a p-type channel, the *ΔV*_T_ in the positive direction compared to the value before illumination suggests the occurrence of an electron-trap effect.^[1,2]^

Following the verification of charge trapping, a more detailed analysis was conducted on the relationship between light intensity and photoconductance. As the light intensity gradually increased, the photoconductance generated in the device also increased, and the data were plotted on a log-log scale (**Figure S6b, Supporting Information**). We performed data fitting using the equation *I*_ph_*=A∙P^α^*, where *I*_ph_ is the photoconductance, *A* is a constant, *P* is the light intensity, and *α* is the fitting parameter. The conduction mechanism can be determined based on the value of *α*: when *α*  = 1, the behavior is fully governed by the photoconductive effect, whereas *α* < 1 indicates the dominance of the photogating effect.^[3,4]^ For the FGPT device, the *α* values were fitted to about 0.18 and 0.59, indicating that two distinct *α* values were obtained. With increasing light intensity, the trap sites gradually become saturated, eventually leading to the generation of untrapped free carriers upon complete saturation.^[5]^ As a result, the photoconductive effect gradually becomes dominant, causing the *α* value to approach 1. The FGPT device also exhibits an increase in *α* as a result of trap saturation. However, the observed value of 0.59 indicates that the photogating effect remains dominant within our measurement range. Such dominance of the photogating effect allows the photoconductance to persist for a long time even after the light is turned off, depending on the lifetime of the trapped photocarriers.^[2]^

**Supplementary Note 2. Synaptic parameters from electrical LTP and LTD**

Neuromorphic behavior is governed by long-term potentiation and depression (LTP/LTD). With repeated stimuli, LTP strengthens synaptic weight and increases conductance, while LTD weakens it under prolonged weak stimuli and reduces conductance. These changes are quantified by nonlinearity ($NL$) and symmetry. $NL$ measures deviations from linear updates, while symmetry reflects the balance between potentiation and depression. Optimized $NL$ and symmetry stabilize weight updates enhance synaptic adaptability, and prevent biased learning that degrades memory retention and recognition accuracy.

**Nonlinearity**

The conductance variation with the number of pulses ($P$) exhibits a nonlinear weight update, evaluated using the following equations:

$G_{\mathrm{LTP}}=B\cdot(1-exp(-P/A))+G_{\min}$ (1)

$G_{\mathrm{LTD}}=-B\cdot(1-exp(P-P_{\max}/A))+G_{\max}$ (2)

$B=(G_{\max}-G_{\min})/(1-exp({-P}_{\max}/A))$ (3)

Here, $G_{\mathrm{LTP}}$ and $G_{\mathrm{LTD}}$​ denote the conductance in the LTP and LTD regions, while $G_{\min}$​ and $G_{\max}$​ represent the minimum and maximum conductance values. The parameter $A$ determines the extent of nonlinear behavior, while fitting constant $B$ normalizes the conductance range relative to $A$, with $NL$ obtained by adjusting $A$. The $A$ value was extracted from experimental data using open-source MATLAB code, and the corresponding $NL$ values were derived from reference tables provided by the same source.^[6]^

**Symmetricity in weight updates**

The symmetricity of the conductance is evaluated by quantifying the relationship between LTP and LTD behaviors. It is defined as the inverse of the symmetric error (*Sym.Err*), calculated using the following equations:

$Sym. Err=\sum_{k=1}^{k=n} \frac{({G_{N}(k)-G_{N}(2n-k))}^{2}}{n}=\sum_{k=1}^{k=n} \frac{(({G\left( k \right)-G_{m\mathrm{in}})-(G(2n-k)-G_{m\mathrm{in}}))}^{2}}{n{(G_{m\mathrm{ax}}-G_{m\mathrm{in}})}^{2}}$

$=\sum_{k=1}^{k=n} \frac{(({G\left( k \right)-G_{m\mathrm{in}})-(G(2n-k)-G_{m\mathrm{in}}))}^{2}}{n{(G_{m\mathrm{ax}}-G_{m\mathrm{in}})}^{2}}=\sum_{k=1}^{k=n} \frac{({G\left( k \right)-G(2n-k))}^{2}}{n{(G_{m\mathrm{ax}}-G_{m\mathrm{in}})}^{2}}$ (4)

where

$G_{N}\left( k \right)=\frac{G\left( k \right)-G_{m\mathrm{in}}}{G_{m\mathrm{ax}}-G_{m\mathrm{in}}}$ (5)

Here, $G\left( k \right)$ and $G\left( 2n-k \right)$ denote conductance for potentiation and depression, respectively, while $G_{N}\left( k \right)$ indicates the normalized conductance at the *k*^th^ state. The symmetricity parameter provides the efficiency and reliability of synaptic weight modulation. Ideally, LTP and LTD curves exhibit perfect symmetry, resulting in a symmetric error value of zero. In contrast, extreme asymmetry leads to an infinite symmetric error, where the difference between potentiation and depression conductance reaches its maximum.

**Symmetry analysis with asymmetric ratio and nonlinearity**

The asymmetric ratio ($AR$) quantifies the balance between LTP and LTD conductance changes, serving as a crucial parameter for assessing synaptic modulation symmetry:

$AR=\frac{\max|G_{\mathrm{LTP}}\left( k \right)-G_{\mathrm{LTD}}\left( k \right)|}{G_{\mathrm{LTP}}\left( n \right)-G_{\mathrm{LTD}}\left( n \right)}$ (6)

Here, $G_{\mathrm{LTP}}\left( k \right)$ and $G_{\mathrm{LTD}}\left( k \right)$ are potentiation and depression conductance values at the *k*^th^ state, while $G_{\mathrm{LTP}}\left( n \right)$ and $G_{\mathrm{LTD}}\left( n \right)$ represent their final values after *n* updates. An ideal synaptic device achieves an $AR$ close to zero, reflecting symmetric and stable conductance changes. Symmetry deviations arise from ionic drift, conductance variability, or non-uniform spike parameters, which can disrupt weight updates and affect learning performance.

$AR$ requires detailed intermediate conductance states, which may not always be available. To address this limitation, $NL$ of LTP and LTD serves as an alternative symmetry metric:

$Symmetry metric=|{NL}_{\mathrm{LTP}}-{NL}_{\mathrm{LTD}}|$ (7)

This metric quantifies the curvature difference between LTP and LTD processes, assuming identical $G_{m\mathrm{in}}$ and $G_{m\mathrm{ax}}$. While practical for simplified analysis, differences in $G_{m\mathrm{in}}$ and $G_{m\mathrm{ax}}$​ between processes may introduce inaccuracies, particularly in systems with large conductance variability. Combining $AR$ and $NL$-based symmetry metrics can comprehensively evaluate synaptic behavior. $AR$ provides precise symmetry analysis when intermediate conductance data are available, while the $NL$-based simplified comparison metric offers a practical alternative for data-limited studies. These approaches provide valuable insights into the performance and reliability of synaptic devices.

**Supplementary Note 3. Crystalline structure and poling current evolution in P(VDF-TrFE) with annealing temperature**

The ferroelectric properties of P(VDF-TrFE) depend on the annealing temperature, which determines the formation of crystalline phases (*α*, *β*, *γ*) and their structural characteristics. Annealing near the Curie temperature (*T*_C_ ~ 140°C) promotes the *β*-phase transition,^[7]^ where the highly ordered all-trans (TTTT) conformation enhances remnant polarization.^[8]^ To verify this, we analyzed the crystalline structure and polarization switching of P(VDF-TrFE) at different annealing temperatures.

The X-ray diffraction (XRD) analysis (**Supplementary Figure S11a)** reveals phase transitions in P(VDF-TrFE). At 25 °C and 80 °C, the diffraction peak at 19.8° (2*θ*) has low intensity, indicating insufficient *β*-phase formation. Instead, it mainly consists of the *α*-phase, which has disordered dipole alignment and poor remnant polarization. Annealing at 130–140 °C enhances *β*-phase formation, as indicated by the strong diffraction peak near 19.8°, corresponding to the (110)/(200) planes. However, above 150 °C, *β*-phase intensity decreases due to structural degradation and phase transitions, which degrade ferroelectric properties. To evaluate the impact of annealing, voltage sweeps from –20 V to 20 V were applied to Au/P(VDF-TrFE)/Pt metal-ferroelectric-metal (MFM) structures. The poling current shows distinct differences between devices annealed at 80 °C and 140 °C (**Supplementary Figure S11b**). At 80 °C, only slight changes occur near the coercive voltage, indicating weak polarization switching and insufficient *β*-phase. In contrast, at 140 °C, a significant increase in poling current near the coercive voltage confirms effective polarization switching, driven by a fully developed *β*-phase.

XRD confirms the crystalline phase transition, while poling current measurements reveal changes in polarization switching behavior as a function of annealing temperature. Optimized *β*-phase formation at 140 °C enables efficient polarization switching, which is crucial for the non-volatile characteristics of the FGPT. In contrast, devices annealed at 80 °C exhibit limited ferroelectric response with minimal potentiation and depression effects. These findings highlight the importance of precise annealing control in achieving strong ferroelectric properties, which are essential for linear multilevel modulation and neuromorphic in-sensor processing.

**Supplementary Note 4. In-sensor processing simulation for all-day face recognition**

The proposed all-day face recognition system demonstrates the capability of FGPT to recalibrate unstructured visual data into pre-trained ranges without retraining or data reconstruction. Utilizing a 64×64 FGPT synaptic array, light information is converted into non-volatile memory currents, allowing independent potentiation, depression, and erasure of stored visual information. This approach ensures robust recognition through in-sensor processing without additional computational resources. The simulation follows three steps: (i) training data processing, (ii) test data processing with electrical processing, and (iii) recognition. The following sections detail each step.

**(i) Training data processing**

During training, input images were resized, normalized, and mapped to a 64×64 synaptic array. Grayscale values were converted to absolute light intensity (*P*_light_) to match the device response range. Memory currents were modeled using quadratic fitting to establish a consistent relationship with light signal. Derived coefficients enabled the precise conversion of light intensity to memory currents, forming the initial memory map. Iterative updates highlighted high-memory current regions, constructing a high-current map by emphasizing the top 75%, 50%, and 25% intensities to enhance feature extraction (**Supplementary Figure S20)**.

**(ii) Test data and electrical processing**

Each test image was converted from grayscale to light intensity and mapped to memory current. Currents outside the predefined training range were adjusted—potentiated if below and depressed if above—aligning each pixel with the trained memory distribution (**Supplementary Figure S21)**. A processing map derived from device measurements determined precise gate voltage for adjustment. This model-based approach, utilizing selective voltage pulses, enables in-sensor neuromorphic processing while minimizing external computation. Fixed training ranges and feature masks, derived from the training data of a specific individual, ensured efficiency. Normalizing test memory maps within the device maintained stable current distributions across varied lighting conditions.

**(iii) Recognition**

An activation threshold determined the number of active pixels in each test image, enabling precise comparison between training and testing memory maps. Pixels were activated if their current matched the feature mask values within a specific tolerance (7%), optimized to minimize noise-related errors across facial orientations and lighting conditions. Test data was classified as a match if 40% or more pixels exceeded this activation threshold. A color-coded image highlighted active regions: background (blue), non-activated (navy), and activated (yellow) (**Supplementary Figure S22)**. This visualization enhanced feature detection and demonstrated accurate face recognition across diverse illumination conditions, consistently distinguishing between matching and non-matching subjects.

This system ensures adaptable face recognition across diverse lighting conditions without advanced post-processing. Unlike conventional in-sensor systems requiring data erasure before re-learning, FGPT enables dynamic test data adjustments, ensuring efficient and sustainable updates. Integrated voltage control allows precise pixel-level tuning while minimizing external processing. This enables real-time, low-power in-sensor processing and enhances scalability for practical applications.

**Supplementary Note 5. Neural network simulation of FGPT-based in-sensor preprocessing for image recognition under variable lighting condition**

To validate the utility of the FGPT as an in-sensor processor for contrast adaptation under variable lighting, a neural network simulation was conducted using the Fashion-MNIST dataset. This dataset, consisting of 28×28 grayscale images across ten fashion categories, is suitable for evaluating classification robustness under various illumination conditions.

The preprocessing steps were as follows. Input images were first converted into light intensity format, considering three lighting conditions: bright, indoor, and dim. They were then transformed into memory maps using a polynomial fit between light intensity and stored current. To evaluate the effect of in-sensor processing enabled by the FGPT, a total of six cases before and after processing under each lighting condition were tested. The simulation used the full Fashion-MNIST test set of 10,000 images for evaluation. For training, only 20% of the original 60,000 training images (i.e., 12,000 samples) were used, and only the normalized memory-mapped input under indoor lighting was included in training. A convolutional neural network (CNN) was employed to evaluate classification performance. The model included two convolutional layers (3×3 filters with 16 and 32 channels), max pooling layers (2×2), and two fully connected layers (32-unit ReLU and 10-unit softmax).

The results showed that under extreme lighting conditions, classification accuracy significantly declined. In contrast, in-sensor processing using the FGPT effectively restored accuracy to levels comparable to those under normal illumination. These findings demonstrate that the FGPT enables hardware-level image recognition without additional model retraining. Moreover, its compatibility with conventional CNNs underscores its potential for scalable neuromorphic vision systems, combining sensing, memory, and processing at the pixel level.

**Supplementary Figures**

**Supplementary Figure S1 | Structural analysis of the 8×8 FGPT array. a,** Scanning transmission electron microscope (STEM) images of the cross-section view for the ferroelectric-gated phototransistor (FGPT) (left). The enlarged image confirms the thickness of the polyvinylidene fluoride-trifluoroethylene (P(VDF-TrFE)) and poly(3-hexylthiophene) (P3HT) layers (right). **b,** Energy-dispersive X-ray spectroscopy (EDS) elemental maps for Au, S, F, Al, and Si.

**Supplementary Figure S2 | Electrical and optical characteristics of FGPT. a,** Transfer characteristics of FGPT measured at drain voltage (*V*_D_) of –5 V. Gate voltage (*V*_G_) sweep ranges are ±1 V, ±3 V, ±5 V, ±7 V, ±9 V, and ±10 V, respectively. **b,** The memory window increases incrementally with *V*_G_ sweep ranges, demonstrating electrical non-volatile characteristics. **c,** Light-response characteristics of FGPT, showing the transfer curve measured under a light intensity (*P*_light_) of 324 W/m^2^ and in dark conditions with the *V*_G_ sweep from +5 V to –5 V.

**Supplementary Figure S3 | Absorption spectrum of the P3HT and photonic response of the FGPT.** **a**, Normalized absorption spectrum of the P3HT film, exhibiting strong absorption across the visible range (400–650 nm). **b,** Light pulse response of the FGPT at 50 Hz, demonstrating nonlinear temporal summation behavior (left). The enlarged image shows temporal differentiation capability by distinguishing at a pulse width (*W*_light_) of 10 ms (right).

**Supplementary Figure S4 | Dynamic range characteristics of the FGPT. a,** Transient and memory currents as a function of *P*_light_ with fitted curves on a logarithmic scale, demonstrating the ability to operate over a wide range of illumination levels. **b,** Comparison of the FGPT dynamic range with other vision systems, including high dynamic range (HDR), low dynamic range (LDR), digital cameras (D-Cam), simultaneous human vision system (S-HVS), and human vision system (HVS). The dynamic range is defined as ${Luminance}_{\max}/{Luminance}_{\min}$, showing that the FGPT exhibits a broader dynamic range compared to conventional vision systems.

**Supplementary Figure S5 | Reliability evaluation of the FGPT over seven days.** Drain current measurements were recorded over one week, showing transient current (*I*_T_) and memory current (*I*_M_). The results demonstrate stable photonic non-volatile characteristics and memory retention, highlighting the long-term reliability under sustained operation.

**Supplementary Figure S6 | Transfer characteristics and power-law equation fitting of FGPT device. a,** Transfer curves of the FGPT device under light illumination (*P*_light_ = 1905 W/m^2^) and dark conditions. The drain voltage (*V*_D_) was fixed at –5 V, and the gate voltage (*V*_G_) was swept from –5 V to +5 V. **b**, The photoconductance (*I*_ph_) data as a function of light intensity were fitted to the power-law equation *I*_ph_=*A∙P^α^*. The fitting resulted in two *α* values of approximately 0.18 and 0.59.

**Supplementary Figure S7 | Comparison of photonic non-volatile and decaying characteristics.** All devices operated under uniform illumination (*P*_light_ = 324 W/m^2^) for 200 s, followed by 300 s in darkness. The tested devices include: (1) FGPT, (2) organic thin-film transistor (OTFT) with a SiO_2_ gate insulator (Au/P3HT/SiO_2_/p^++^-Si), and (3) Metal-semiconductor-metal (MSM) with Au/P3HT/Al structure.

**Supplementary Figure S8 | Photonic non-volatile property evaluation based on exponential fitting analysis. a**, Photocurrent decay curves after light-off under various illumination intensities, along with the extracted $\tau_{2}$ values. **b**, Extracted $\tau_{2}$ values as a function of illumination intensity, along with the corresponding exponential fitting curves.

**Supplementary Figure S9 | LTP/LTD characteristics and synaptic parameters under various *V*_D_.** Long-term potentiation (LTP) and long-term depression (LTD) behavior of the FGPT confirm partial polarization switching. Each dataset includes 200 potentiation and 200 depression pulses across two cycles, under non-identical amplitude *V*_G_ pulses (1 V → –6 V → 1 V). The table summarizes synaptic parameters, including symmetric error (*Sym.Err*) and nonlinearity (*NL*_LTP_ and *NL*_LTD_).

**Supplementary Figure S10** | **Energy band diagrams and carrier dynamics in the FGPT. a,** Initial energy band structure of the FGPT under dark conditions, along with the energy level table of each material. **b,**Energy band modulation under light and electrical stimulation. Under illumination, the photogating effect occurs via electron–hole pair generation and interfacial electron trapping. Subsequent potentiation and depression pulses induce partial polarization switching, leading to ferroelectric gating-dominant states, which result in hole accumulation and depletion. **c,** Schematic illustration of electron and hole dynamics under photogating-dominant and ferroelectric-gating-dominant conditions.

**Supplementary Figure S11 | Ferroelectric integrity of P(VDF-TrFE). a,** XRD patterns of P(VDF-TrFE) films annealed from 25 °C to 170 °C, showing *β*-phase evolution at 2θ = 19.8°. The *β*-phase forms near 140 °C and degrades above 150 °C. **b,** Poling current of Au/P(VDF-TrFE)/Pt MFM structures. Weak switching at 80 °C contrasts with clear coercive peaks at 140 °C, confirming effective polarization. **c,** XRD and P-E loops after post-annealing. Stable polarization is retained after annealing at 140 °C and at 140 °C followed by 120 °C, but degraded after annealing at 140 °C followed by 160 °C. This confirms 120 °C post-annealing does not impair ferroelectricity.

**Supplementary Figure S12 | Photonic and electrical volatile characteristics in the OTFT.** Current behavior under potentiation (left) and depression (right) *V*_G_ input. The OTFT exhibits volatile photocurrent with no response to electrical pulses (*P*_light_ = 324 W/m²).

**Supplementary Figure S13 | Electrical post-processing for memory current modulation. a,** *ΔI*_D_ response to identical *V*_G_ (< –5 V) pulses during potentiation processing. The memory current increases from the minimum *I*_M_ states (0.025 nA) to the maximum of dynamic range (~ 770 nA). **b,** *ΔI*_D_ response to identical *V*_G_ (> –5 V) pulses during depression processing. The memory current decreases from the maximum *I*_M_ states (470 nA) to the minimum of dynamic range (0 nA), covering the full modulation range. **c–d**, Memory state modulation after illumination exhibits approximately 32 discrete levels, confirming non-volatile and reconfigurable conductance control.

**Supplementary Figure S14 |** **Structural design and measurement setup of the 8×8 FGPT array. a,** Optical image of fabricated 8×8 FGPT array (top-view). **b,** Optical microscopy (OM) image confirming the array design, showing a channel length of 100 μm and a channel width of 1000 μm. **c,** Measurement setup of the 8×8 FGPT array.

**Supplementary Figure S15 | Statistical distribution of drain current change (*ΔI*_D_) under depression and potentiation pulse operations.** Each box-and-whisker plot represents the distribution of *ΔI*_D_ across 19 devices for each pulse number, measured during depression and potentiation operations under pulsed gate voltages.

**Supplementary Figure S16 | Measurement of 8×8 FGPT array under light illumination and electrical post-processing.** Photocurrent data from the 8×8 FGPT array demonstrate the formation of 'GIST' pattern and the contrast enhancement via electrical processing. The pattern appears under low-intensity illumination (127 W/m^2^) against a high-intensity background (536 W/m^2^).

**Supplementary Figure S17 | Memory current characteristics across light intensities. a,** Linear-scale curve of *I*_M_ as a function of light intensity, demonstrating stable performance under different illumination conditions. The fitted curves and corresponding parameters are shown.

**Supplementary Figure S18 | Electrical pulse response under different light intensities. a,** *ΔI*_D_ in response to identical *V*_G_ pulses shows consistent current response under various lighting conditions (*P*_light_ = 5.08 W/m^2^, 127 W/m^2^, 536 W/m^2^, 1905 W/m^2^). **b,** The enlarged view from 310 s to 410 s demonstrates identical potentiation behavior regardless of initial light intensity.

.

**Supplementary Figure S19 | Electrical processing curves for potentiation and depression. a,** Fitted potentiation curve shows –*ΔI*_D_ responses under gate voltage amplitude (*ΔV*_G_) ranging from 0 to –5 V. **b,** Fitted depression curve represents –*ΔI*_D_ responses under *ΔV*_G_ ranging from 0 to 17 V. The fitted curve is based on experimental data, with the fitting parameters shown in **Supplementary Table S4.**

**Supplementary Figure S20 | Workflow for training data processing.** Light intensity (*P*_light_) from the training face is matched to memory currents, generating a memory map. This map is used to define the training range and generate feature masks, highlighting high-memory regions to enhance feature representation for recognition.

**Supplementary Figure S21 | Electrical processing of test data in unstructured environments.** Test faces under unstructured light conditions (e.g., dim or bright) are processed to generate memory maps. Potentiation or depression pulses adjust memory currents to align with the predefined training range, enabling effective contrast enhancement.

**Supplementary Figure S22 | Recognition process and tolerance optimization.** **a**, The test memory current map is compared with the training feature mask to identify activated (yellow) and non-activated (navy) pixels. Activation rates are calculated to enable recognition by quantifying the match between testing and training data. **b,** Activation rates of trained and untrained subjects are plotted against tolerance thresholds. The difference in activation rate (*ΔAR*) between trained and untrained cases peaks at ±7%, indicating this threshold provides the most reliable discrimination by minimizing false rejection and false acceptance.

**Supplementary Figure S23 | Training and testing with trained data under structured lighting conditions. a,** Nine face images of a single individual are sequentially illuminated to the FGPT, updating the memory current map. **b,** Feature masks are derived from the final memory current map, representing the top 100%, 75%, 50%, and 25% of the current values. **c,** Activation rate (A.R.) for test data under different lighting conditions (indoor, dim, bright), evaluated before and after processing, shows an enhancement in the activation rate.

**Supplementary Figure S24 | Activation rate for the untrained dataset.** Comparison of the memory map, memory current histogram, and activation rate before and after processing. The untrained dataset exhibits low activation rates with post-processing, demonstrating reliability in distinguishing untrained data.

**Supplementary Figure S25 | Training and testing with trained data under unstructured lighting conditions. a,** Nine face images of a single individual are sequentially illuminated to the FGPT, updating the memory current map. **b,** Feature masks are derived from the final memory current map, representing the top 100%, 75%, 50%, and 25% of the current values. **c,** Activation rate (A.R.) for test data under different lighting conditions (indoor, dim, bright), evaluated before and after processing, shows an enhancement in the activation rate and confirms the reliability of the process.

**Supplementary Figure S26 | Neural network simulation of FGPT-based in-sensor preprocessing for image recognition. a,** Schematic of FGPT-based preprocessing under different illumination conditions. **b,** Fashion-MNIST images under bright, indoor and dim lighting. **c,** Comparison of input dataset before and after FGPT-based in-sensor processing under each lighting condition. **d,** CNN architecture for 28 × 28 image classification across 10 categories. **e,** Accuracy comparison across 50 epochs under three lighting conditions, before and after in-sensor preprocessing. **f,** Confusion matrices after FGPT processing under bright and dim condition, demonstrating high recognition accuracy.

**Supplementary Tables**

**Supplementary Table S1 |** Comparison of processing mechanisms in optoelectronic synaptic devices.

| **Device type** | **Weight modulation mechanism** | **Wavelength** | **Operating voltage** | **Application** | **Ref** |
| --- | --- | --- | --- | --- | --- |
| RRAM | Optical/Electrical writing  ↔ Optical/Electrical erasing | 405 nm | < 1V | Contrast enhancement | [9] |
| Two-terminal  optoelectronic  synapse | Optical writing  → Optical erasing | 400–800 nm | 2 V | Face  recognition | [10] |
| Opto-electrochemical transistor | Optical writing  → Electrical erasing | 400–700 nm | < 1 V | Face  recognition | [11] |
| Phototransistor | Optical writing  → Optical erasing | 365–633 nm | ~ 30 V | Spectral adaptation | [12] |
|  | Optical writing  → Relaxation | 365 nm  / 520 nm  / 650 nm | ~ 10 V | Image  recognition | [13] |
|  | Optical writing  → Optical erasing | 405–940 nm | 25 V | Motion  detection | [14] |
|  | Optical writing  → Optical erasing | 450 nm  / 520 nm  / 637 nm | ~ 14 V | Motion  detection | [15] |
|  | Optical writing  → Electrical erasing | White light | 1 V | Image  processing | [16] |
| **Ferroelectric phototransistor** | **Optical writing**  **→ Post-processing**  **(Electrical pot. / dep.)** | **Visible**  **(300–700 nm)** | **~ 5 V** | **All-day face recognition** | **This**  **work** |

**Supplementary Table S2 |** Extracted FET parameters under dark conditions.

| **Parameter** | **Equation** | **Forward**  **(5V 🡪 –5V)** | **Reverse**  **(–5V 🡪 5V)** |
| --- | --- | --- | --- |
| **On/Off Ratio** | $\frac{I_{\mathrm{ON}}}{I_{\mathrm{OFF}}}$ | 49.63 | |
| **Mobility (cm^2^/V∙s)** | $\mu= \frac{2L}{WC_{i}}\left( \frac{\partial\sqrt{I_{\mathrm{DS}}}}{\partial V_{G}} \right)^{2}$ | 0.02590 | 0.0261 |
| **Subthreshold Swing (V/dec)** | $SS= \frac{dV_{\mathrm{GS}}}{d\left( \log I_{\mathrm{DS}} \right)}$ | 4.684 | 4.247 |

**Supplementary Table S3 |** Comparison of PNV coefficient in synaptic devices.

| **Structure** | **Response wavelength (nm)** | **Pulse width**  **(Optical)** | **PNV Coefficient** | **Ref.** |
| --- | --- | --- | --- | --- |
| 4H-SiC / PVK / P3HT Transistor | UV | 50 ms | 35% | [4] |
| P3HT:PCBM OECT | Visible | 40 ms | 50% | [11] |
| P(VDF-TrFE) / MoTe_2_ Homojunction | UV – NIR | 1 s | 0% | [17] |
| Si / SiO_2_ / CDs-Silk / Pentacene Transistor | UV | 1s | 45% | [18] |
| ReS_2_ FeFET | Visible | 100 ms | 58% | [19] |
| ITO / P3HT / Al | Visible | 1 s | 20% | [20] |
| MoS_2_ / Ba_0.6_Sr_0.4_TiO_3_  FeFET | Visible –NIR | 0.5 s | 45% | [21] |
| CsPbBr₃ QDs / PMMA / Pentacene FET | Visible | 1 s | 80% | [22] |
| WS_2_ / PZT FeFET. | Visible | 100 ms | 59% | [23] |
| Black Phosphorus (BP) Transistor | UV | 1 ms | 16% | [24] |
| Pd / MoOₓ / ITO  ORRAM | UV | 200 ms | 12% | [25] |
| ITO / Nb:SrTiO_3_ Heterojunction | Visible | 500 ms | 69% | [26] |
| **P3HT / P(VDF-TrFE) FeFET** | **Visible** | **5 ms** | **77%** | **This work** |

* PNV coefficient = *I*ₘₑₘₒᵣᵧ_,3s_ / *I*ₜᵣₐₙₛᵢₑₙₜ, where *I*ₜᵣₐₙₛᵢₑₙₜ is the peak current immediately after a minimal-width light pulse, and *I*ₘₑₘₒᵣᵧ_,3s_ is the current measured after 3 seconds.

**Supplementary Table S4 |** Fitted constants for potentiation and depression processing maps.

| $\boldsymbol{y=B}\boldsymbol{e}^{\frac{\boldsymbol{x}}{\boldsymbol{t}}}\boldsymbol{+}\boldsymbol{y}_{\boldsymbol{0}}$ | | | |
| --- | --- | --- | --- |
| Processing | ***B*** | ***y*_0_** | ***t*** |
| Potentiation *ΔV*_G_  vs –*ΔI*_D_ | 1.23×10^-8^ ± 3.24×10^-9^ | –7.64×10^-9^ ± 4.04×10^-8^ | 0.88 ± 0.04 |
| Depression *ΔV*_G_  vs –*ΔI*_D_ | –1.95×10^-7^ ± 5.48×10^-8^ | 2.02×10^-7^ ± 5.95×10^-8^ | 15.44 ± 2.61 |

**References in Supplementary Information**

[1] Chen, J. Y., Chiu, Y. C., Li, Y. T., Chueh, C. C., & Chen, W. C., Nonvolatile perovskite‐based photomemory with a multilevel memory behavior, 2017, Adv. Mater., 29, 1702217, <https://doi.org/10.1002/adma.201702217>

[2] Feng, G., Zhang, X., Tian, B., & Duan, C., Retinomorphic hardware for in‐sensor computing, 2023, InfoMat, 5, e12473, <https://doi.org/10.1002/inf2.12473>

[3] Li, L., Wang, W., Chai, Y., Li, H., Tian, M., & Zhai, T., Few‐Layered PtS2 Phototransistor on h‐BN with High Gain, 2017, Adv. Funct. Mater., 27, 1701011, <https://doi.org/10.1002/adfm.201701011>

[4] Liu, X., Huang, W., Kai, C., Yin, L., Wang, Y., Liu, X., Pi, X., et al., Photogated synaptic transistors based on the heterostructure of 4H-SiC and organic semiconductors for neuromorphic ultraviolet vision, 2023, ACS Appl. Electron. Mater., 5, 367, <https://doi.org/10.1021/acsaelm.2c01390>

[5] Island, J. O., Blanter, S. I., Buscema, M., van der Zant, H. S., & Castellanos-Gomez, A., Gate Controlled Photocurrent Generation Mechanisms in High-Gain In2Se3 Phototransistors, 2015, Nano Lett., 15, 7853, <https://doi.org/10.1021/acs.nanolett.5b02523>

[6] Chen, P. Y., Peng, X., & Yu, S., NeuroSim: A circuit-level macro model for benchmarking neuro-inspired architectures in online learning, 2018, IEEE Trans. Comput.-Aided Des. Integr. Circuits Syst., 37, 3067, 10.1109/TCAD.2018.2789723

[7] Kim, S., Heo, K., Lee, S., Seo, S., Kim, H., Cho, J., Lee, H., et al., Ferroelectric polymer-based artificial synapse for neuromorphic computing, 2021, Nanoscale Horiz., 6, 139, <https://doi.org/10.1039/D0NH00559B>

[8] Ahmed, A., Jia, Y., Deb, H., Arain, M. F., Memon, H., Pasha, K., Huang, Y., et al., Ultra-sensitive all organic PVDF-TrFE E-spun nanofibers with enhanced *β*-phase for piezoelectric response, 2022, J. Mater. Sci.: Mater. Electron., 33, 3965, <https://doi.org/10.1007/s10854-021-07590-y>

[9] Zhou, G., Li, J., Song, Q., Wang, L., Ren, Z., Sun, B., Hu, X., et al., Full hardware implementation of neuromorphic visual system based on multimodal optoelectronic resistive memory arrays for versatile image processing, 2023, Nat. Commun., 14, 8489, <https://doi.org/10.1038/s41467-023-43944-2>

[10] Jeon, Y., Lee, G., Kim, Y. J., Jang, B. C., & Yoo, H., Dual Synapses and Security Devices from Ternary C60‐Pentacene‐TiO2‐x Nanorods Heterostructures, 2024, Adv. Funct. Mater., 34, 2409578, <https://doi.org/10.1002/adfm.202409578>

[11] Chen, K., Hu, H., Song, I., Gobeze, H. B., Lee, W. J., Abtahi, A., Schanze, K. S., et al., Organic optoelectronic synapse based on photon-modulated electrochemical doping, 2023, Nat. Photonics, 17, 629, <https://doi.org/10.1038/s41566-023-01232-x>

[12] Wen, W., Liu, G., Wei, X., Huang, H., Wang, C., Zhu, D., Sun, J., et al., Biomimetic nanocluster photoreceptors for adaptative circular polarization vision, 2024, Nat. Commun., 15, 2397, <https://doi.org/10.1038/s41467-024-46646-5>

[13] Yu, R., He, L., Gao, C., Zhang, X., Li, E., Guo, T., Li, W., et al., Programmable ferroelectric bionic vision hardware with selective attention for high-precision image classification. 2022, Nat. Commun., 13, 7019, <https://doi.org/10.1038/s41467-022-34565-2>

[14] Pang, X., Wang, Y., Zhu, Y., Zhang, Z., Xiang, D., Ge, X., Wu, H., et al., Non-volatile rippled-assisted optoelectronic array for all-day motion detection and recognition, 2024, Nat. Commun., 15, 1613, <https://doi.org/10.1038/s41467-024-46050-z>

[15] Zhang, Z., Wang, S., Liu, C., Xie, R., Hu, W., & Zhou, P., All-in-one two-dimensional retinomorphic hardware device for motion detection and recognition, 2022, Nat. Nanotechnol., 17, 27, <https://doi.org/10.1038/s41565-021-01003-1>

[16] Choi, C., Leem, J., Kim, M., Taqieddin, A., Cho, C., Cho, K. W., Lee, G. J., et al., Curved neuromorphic image sensor array using a MoS2-organic heterostructure inspired by the human visual recognition system, 2020, Nat. Commun., 11, 5934, <https://doi.org/10.1038/s41467-020-19806-6>

[17] Wu, G., Zhang, X., Feng, G., Wang, J., Zhou, K., Zeng, J., Dong, D., et al., Ferroelectric-defined reconfigurable homojunctions for in-memory sensing and computing, 2023, Nat. Mater, 22, 1499, <https://doi.org/10.1038/s41563-023-01676-0>

[18] Lv, Z., Chen, M., Qian, F., Roy, V. A., Ye, W., She, D., Wang, Y., et al., Mimicking neuroplasticity in a hybrid biopolymer transistor by dual modes modulation, 2019, Adv. Funct. Mater., 29, 1902374, <https://doi.org/10.1002/adfm.201902374>

[19] Chen, Y., Wang, Z., Du, J., Si, C., Jiang, C., & Yang, S., Wrinkled Rhenium Disulfide for Anisotropic Nonvolatile Memory and Multiple Artificial Neuromorphic Synapses, 2024, ACS Nano, 18, 30871, <https://doi.org/10.1021/acsnano.4c11898>

[20] Qian, Y., Li, J., Shen, J., Ke, Y., Yu, S., Li, W., Xu, X., et al., In‐Depth Physical Mechanism Analysis of Polymer Artificial Optoelectronic Synapse with High Endurance and Applications of Visual System and Operant Conditioning, 2023, Adv. Electron. Mater., 9, 2300135, <https://doi.org/10.1002/aelm.202300135>

[21] Tan, C., Wu, H., Lin, Z., Liu, J., Yang, L., Gao, L., & Wang, Z., Human Memory‐Inspired MoS2/BST Ferroelectric Phototransistor with Synchronous Sensory, Short‐Term and Long‐Term Memories, 2025, Adv. Funct. Mater., 35, 2414186, <https://doi.org/10.1002/adfm.202414186>

[22] Wang, Y., Lv, Z., Chen, J., Wang, Z., Zhou, Y., Zhou, L., Chen, X., et al., Photonic synapses based on inorganic perovskite quantum dots for neuromorphic computing, 2018, Adv. Mater., 30, 1802883, <https://doi.org/10.1002/adma.201802883>

[23] Luo, Z. D., Xia, X., Yang, M. M., Wilson, N. R., Gruverman, A., & Alexe, M., Artificial optoelectronic synapses based on ferroelectric field-effect enabled 2D transition metal dichalcogenide memristive transistors, 2020, ACS Nano, 14, 746, <https://doi.org/10.1021/acsnano.9b07687>

[24] Ahmed, T., Tahir, M., Low, M. X., Ren, Y., Tawfik, S. A., Mayes, E. L., Kuriakose, S., et al., Fully light‐controlled memory and neuromorphic computation in layered black phosphorus, 2021, Adv. Mater., 33, 2004207, <https://doi.org/10.1002/adma.202004207>

[25] Zhou, F., Zhou, Z., Chen, J., Choy, T. H., Wang, J., Zhang, N., Lin, Z., et al., Optoelectronic resistive random access memory for neuromorphic vision sensors. 2019, Nat. Nanotechnol., 14, 776, <https://doi.org/10.1038/s41565-019-0501-3>

[26] Gao, S., Liu, G., Yang, H., Hu, C., Chen, Q., Gong, G., Xue, W., et al., An oxide Schottky junction artificial optoelectronic synapse, 2019, ACS Nano, 13, 2634, <https://doi.org/10.1021/acsnano.9b00340>
